# Supplementary material for: Single-Molecule Unbinding Forces between the Polysaccharide Hyaluronan and Its Binding Proteins
Source: Biophys J. 2018 Jun 19;114(12):2910–22. doi: 10.1016/j.bpj.2018.05.014 (PMC6026378; doi:10.1016/j.bpj.2018.05.014)
Supplement: Document S1. Figs. S1–S6 and Table S1 [file mmc1.pdf]

Biophysical Journal, Volume *114*

Supplemental Information

Single-Molecule Unbinding Forces between the Polysaccharide Hyaluronan and Its Binding Proteins

Fouzia Bano, Markku I. Tammi, David W. Kang, Edward N. Harris, and Ralf P. Richter

SUPPORTING FIGURES

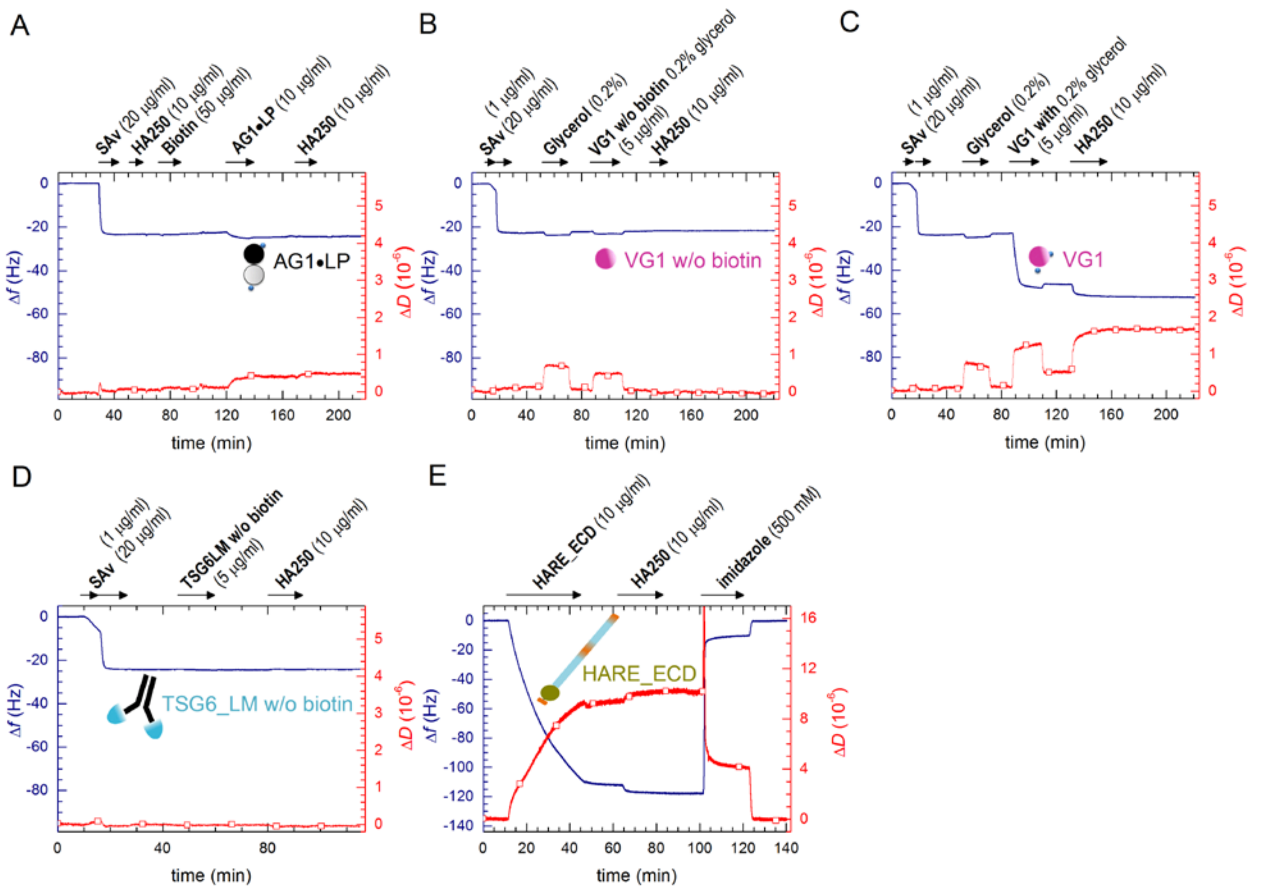

Figure S1: Complementary QCM-D assays demonstrating the specificity of hyaladherin immobilization and HA binding. Data are presented analogous to Fig. 2. (A-D) Streptavidin monolayers were either blocked with free biotin prior to hyaladherin incubation (for AG1•LP; A) or exposed to hyaladherins lacking biotin tags (for VG1 and TSG6_LM; B and D, respectively). In combination with Fig. 2, the QCM-D responses shown here demonstrate non-specific binding of only a minor fraction of AG1•LP (A) and no detectable non-specific binding for VG1 (B) and TSG6_LM (D), and that HA binds exclusively to the specifically immobilized proteins. B and C also contain an incubation step of the streptavidin monolayer with 0.2% glycerol in working buffer (Δf = -1.5 ± 0.4 Hz and ΔD = 0.6 ± 0.1 × 10^{-6}), a concentration that is also contained in the working solutions of incubated VG1. This control demonstrates that the QCM-D responses for VG1 without biotin (B) are due to the presence of glycerol and do not reflect a transient binding, and that the responses for VG1 with biotin (C) are also affected by glycerol. This solution effect has been corrected for in Fig. 2B. (E) HARE_ECD can be fully eluted in imidazole, demonstrating that binding to the His-tag-capturing layer is specific through the His tag. Changes in Δf and ΔD upon exchange from imidazole containing solution to pure working buffer (at 123 min) do not reflect any changes on the surface but result from a change in the viscosity and/or density of the surrounding solution owing to the presence of imidazole. Similarly, the small decrease in dissipation (ΔD = 0.4 ± 0.1 × 10^{-6}) upon exchange from HARE_ECD containing solution to pure working buffer (at 46 min) reflects the effect of residual buffer components in the protein stock solution, and this has been corrected for in Fig. 2D.

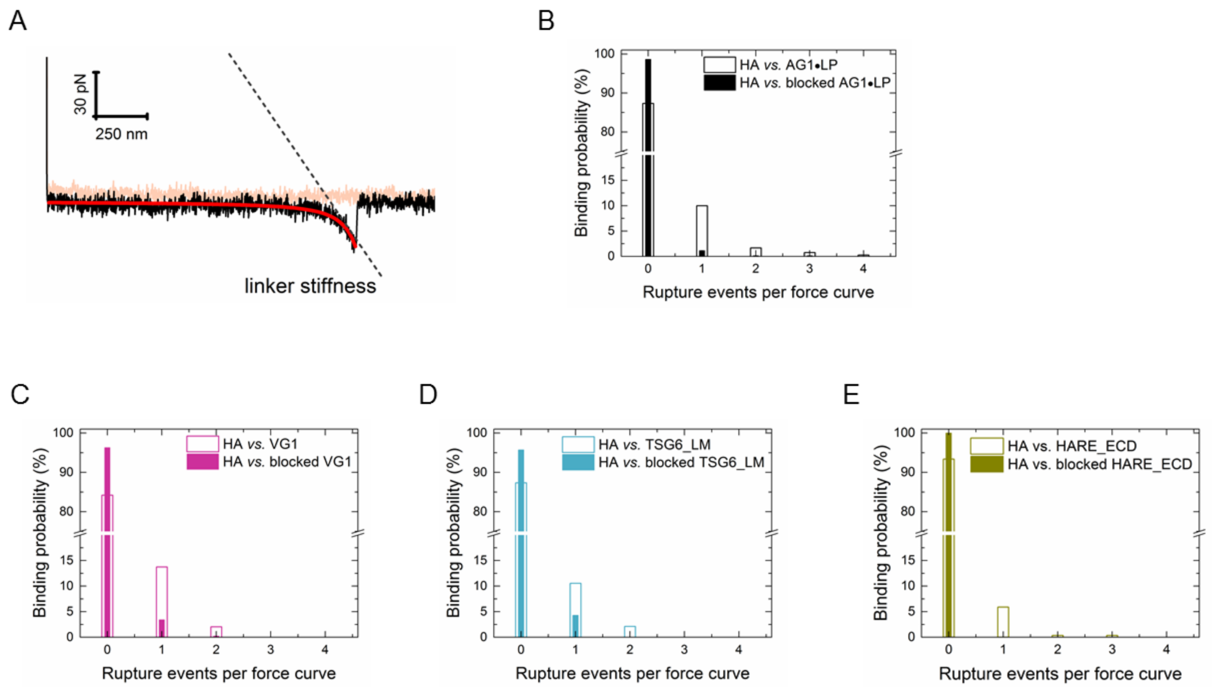

Figure S2: (A) A representative force curve (approach in pink and retract in black) acquired for a specific unbinding event between HA and VG1 (retract velocity 1500 nm/s). The solid red line is a best-fit WLC model curve for stretching a single HA chain. The slope of the dashed straight line represents the linker stiffness (*i.e.*, the slope of WLC fit) at bond rupture, which together with the retract velocity gives the instantaneous loading rate. (B-E) Probability of specific rupture events per force curve for the interactions as indicated in the plots (retract velocities: 2000 nm/s - AG1•LP, 3000 nm/s - VG1, 1000 nm/s - TSG6_LM and HARE_ECD). Competition with a low-molecular-weight HA polymer in solution (58 kDa; 10 µg/ml; filled bars) drastically reduced the occurrence of specific rupture events compared to the equivalent system without the competitor (open bars). The analysis is based on 1200/800 (AG1•LP; B), 342/400 (VG1; C), 237/200 (TSG6_LM; D) and 559/232 (HARE_ECD; E) force curves per sample for the measurements without/with competitor, respectively.

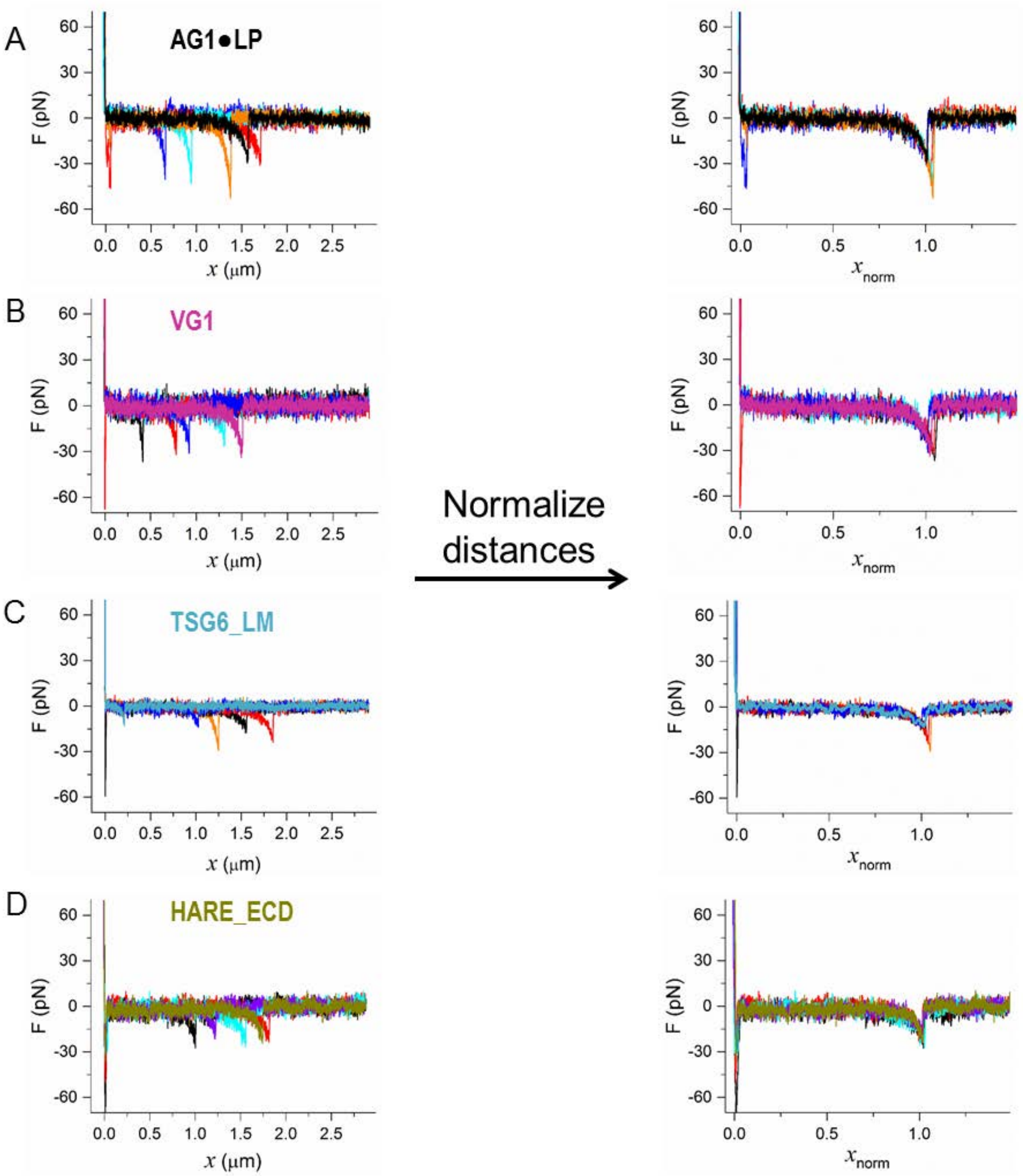

Figure S3: Comparison of force curves by normalization to account for variations in the locus of hyaladherin binding along the HA chain. Shown on the left are sets of 5 force curves per hyaladherin (as indicated; retract velocity 1000 nm/s) featuring a single specific rupture event along with non-specific interactions at very small separations. Curves of force *F vs.* distance *x* were selected at random but with a bias to cover a large spectrum of binding loci on the HA chain; that is, the distances at rupture (ranging from 245 to 1852 nm) span a large part of the HA contour length (2.1 μm). Shown on the right are the same force curves with the distances re-scaled such that $x_{norm} = x/x^*$ with $F(x^*) = F^*$ where the force F^* was set individually for each hyaladherin to be slightly inferior to the rupture forces (AG1•LP – 24 pN, VG1 – 19 pN, TSG6_LM – 10 pN, HARE_ECD – 13 pN). According to the WLC model, parts of the curves prior to the rupture event should overlap if the stretching and unbinding of a single HA chain is consistently being probed (1), and this is indeed the case.

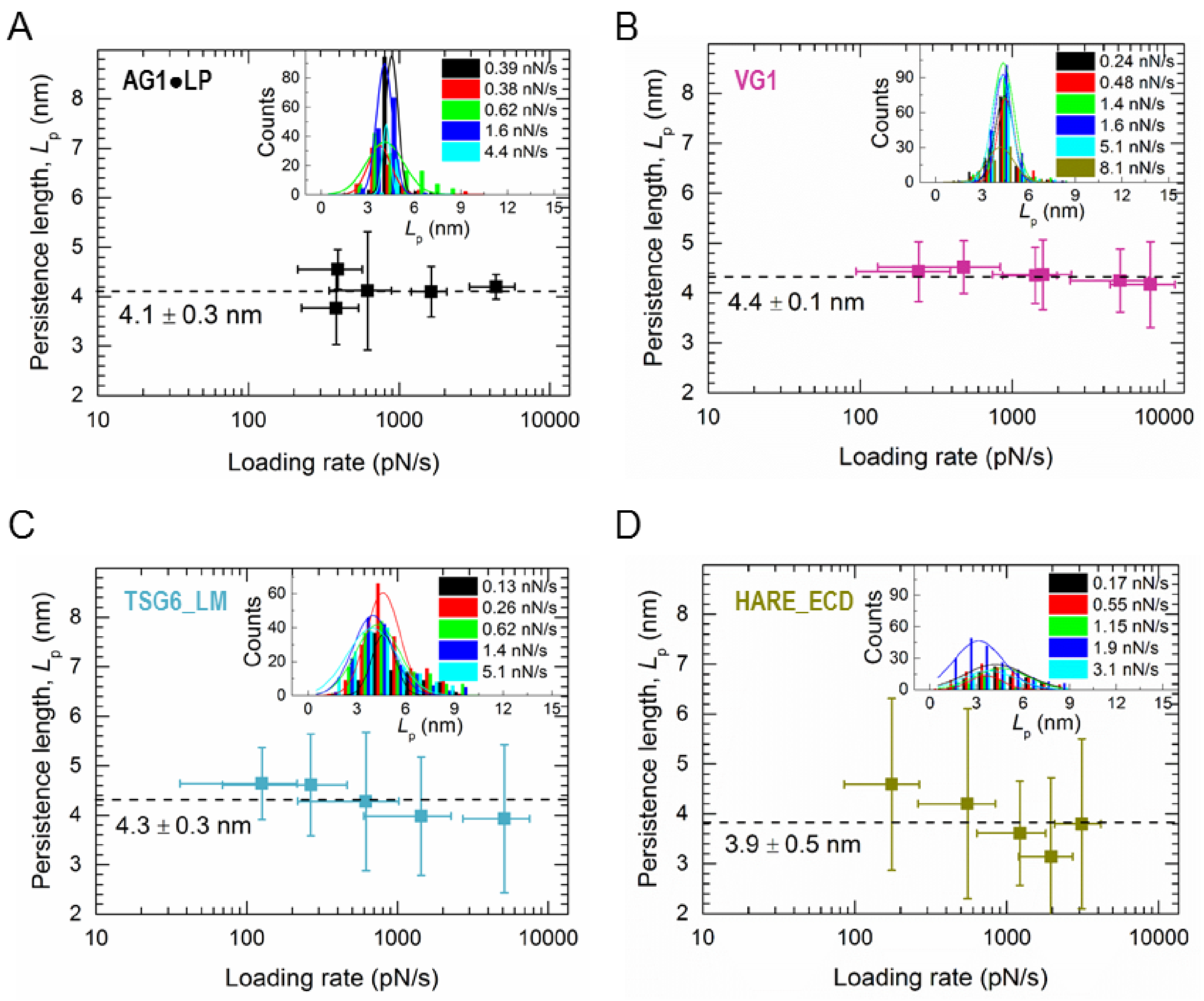

Figure S4: Persistence length (L_p) versus instantaneous loading rates for the data shown in Fig. 4. Dashed horizontal lines represent the mean and numbers indicate the mean ± s.d. The inset shows histograms of L_p for the studied instantaneous loading rates (as listed with color codes) with Gaussian fits from which the mean and standard deviations were calculated. From the four mean values displayed in A to D, a mean persistence length of 4.2 ± 0.2 nm (mean ± s.d.) was extracted.

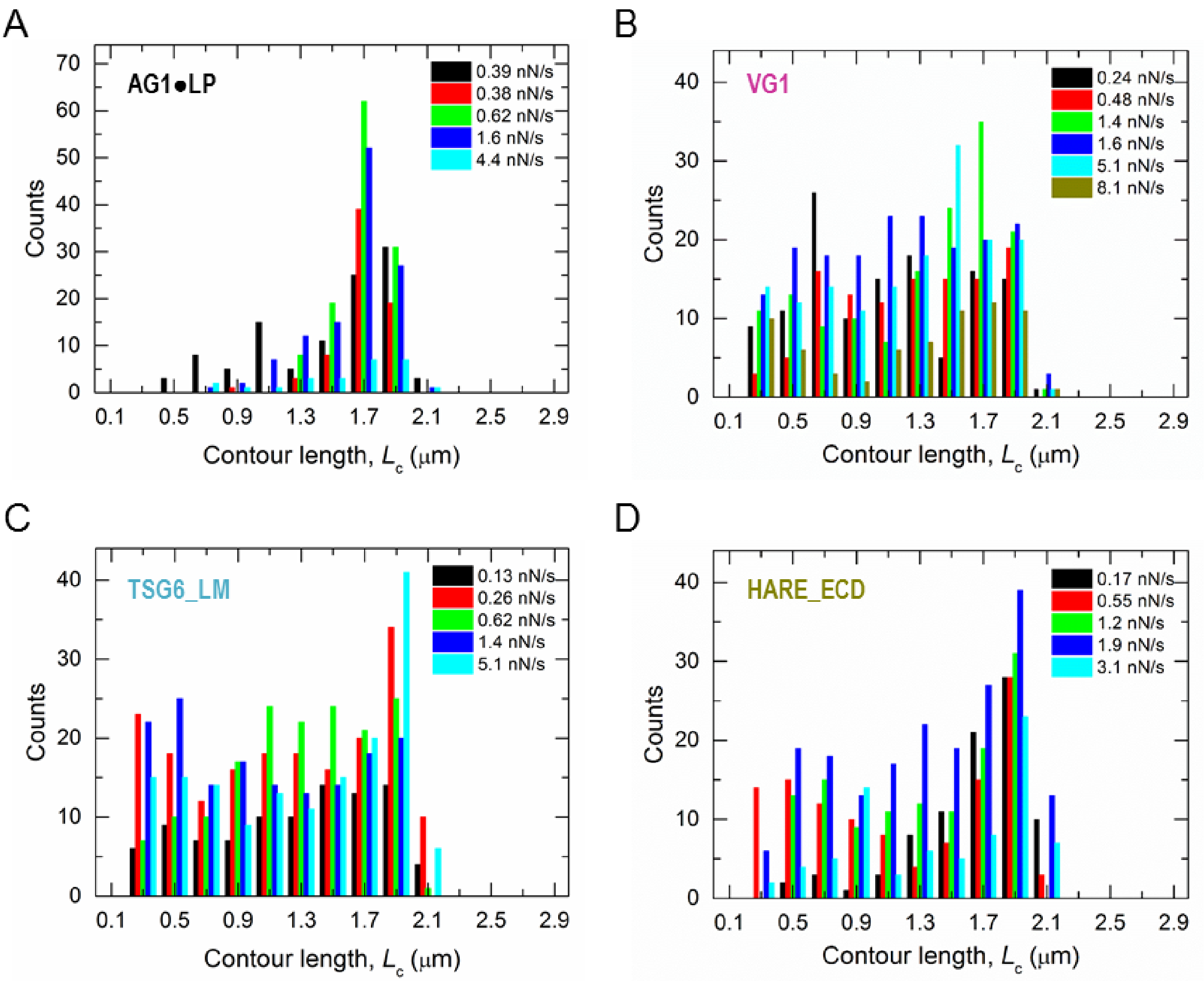

Figure S5: Histograms of contour lengths for the data shown in Fig. 4, displayed by loading rates (as listed with color codes).

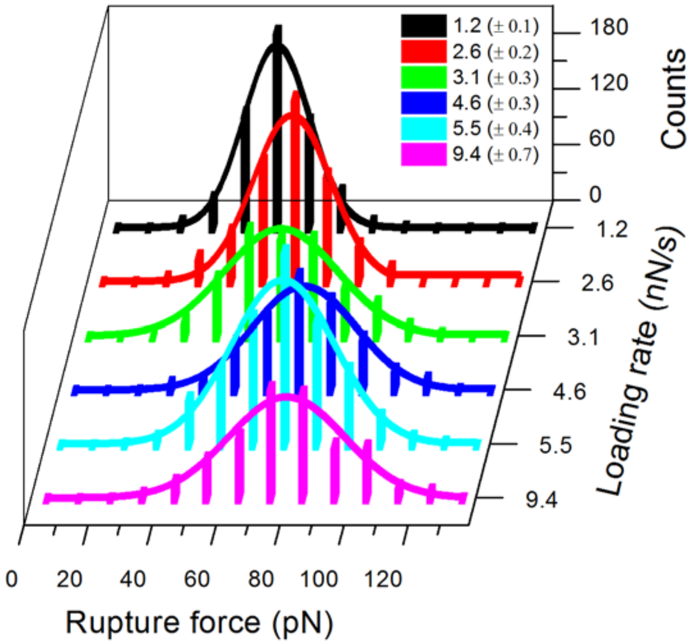

Figure S6: Rupture force histograms for various instantaneous loading rates (listed with color codes as mean ± s.d.) for the interaction of streptavidin and biotin. Solid lines represent Gaussian fits. Data adapted from (2), where streptavidin was immobilized as described in the present work and biotin was tethered *via* a thiol-poly(ethylene glycol) to a gold-coated AFM tip.

SUPPORTING TABLES

Table S1: Overview of force curve sample sizes.

HA binding to	AFM probe	Mean loading rate (nN/s)	Number of force curves			
			Total	Specific rupture events		
				0	1	≥2
AG1•LP	NPG	0.38	3159	2945	106	54
	OBL	0.39	1001	931	58	6
	NPG	0.62	1027	910	90	27
	NPG	1.6	1001	919	50	32
	NPG	4.4	603	577	23	3
VG1	OBL	0.24	708	582	121	5
		0.48	784	644	113	27
		1.4	1367	1182	147	33
		1.6	564	386	145	33
		5.1	903	729	156	18
		8.1	1429	1357	69	3
TSG6_LM	OBL	0.13	778	698	64	16
		0.26	1293	185	127	29
		0.62	757	630	93	34
		1.4	703	572	105	26
		5.1	775	663	97	15
HARE_ECD	OBL	0.17	1442	1355	67	20
		0.55	1001	893	85	23
		1.2	1485	1364	92	29
		1.9	1395	1240	127	28
		3.1	1216	1141	61	14

SUPPORTING REFERENCES

1. Janshoff, A., M. Neitzert, Y. Oberdorfer, and H. Fuchs. 2000. Force Spectroscopy of Molecular Systems-Single Molecule Spectroscopy of Polymers and Biomolecules. Angew. Chem. Int. Ed. Engl. 39:3212-3237.
2. Bano, F., S. Banerji, M. Howarth, D. G. Jackson, and R. P. Richter. 2016. A single molecule assay to probe monovalent and multivalent bonds between hyaluronan and its key leukocyte receptor CD44 under force. Sci. Rep. 6:34176.
