# Supplementary material for: Single-Molecule Unbinding Forces between the Polysaccharide Hyaluronan and Its Binding Proteins
Source: Biophys J. 2018 Jun 19;114(12):2910–22. doi: 10.1016/j.bpj.2018.05.014 (PMC6026378; doi:10.1016/j.bpj.2018.05.014)
Supplement: Document S2. Article plus Supporting Material [file mmc2.pdf]

Biophysical Journal
Article

Biophysical Society

Single-Molecule Unbinding Forces between the Polysaccharide Hyaluronan and Its Binding Proteins

Fouzia Bano,[1,2] Markku I. Tammi,[3] David W. Kang,[4] Edward N. Harris,[5] and Ralf P. Richter[1,2,*]
[1]School of Biomedical Sciences, Faculty of Biological Sciences, School of Physics and Astronomy, Faculty of Mathematics and Physical Sciences, and Astbury Centre for Structural Molecular Biology, University of Leeds, Leeds, United Kingdom; [2]CIC biomaGUNE, Biosurfaces Laboratory, Donostia-San Sebastian, Spain; [3]Institute of Biomedicine, University of Eastern Finland, Kuopio, Finland; [4]Halozyme Therapeutics Inc., San Diego, California; and [5]Department of Biochemistry, University of Nebraska, Lincoln, Nebraska

ABSTRACT The extracellular polysaccharide hyaluronan (HA) is ubiquitous in all vertebrate tissues, where its various functions are encoded in the supramolecular complexes and matrices that it forms with HA-binding proteins (hyaladherins). In tissues, these supramolecular architectures are frequently subjected to mechanical stress, yet how this affects the intermolecular bonding is largely unknown. Here, we used a recently developed single-molecule force spectroscopy platform to analyze and compare the mechanical strength of bonds between HA and a panel of hyaladherins from the Link module superfamily, namely the complex of the proteoglycan aggrecan and cartilage link protein, the proteoglycan versican, the inflammation-associated protein TSG-6, the HA receptor for endocytosis (stabilin-2/HARE), and the HA receptor CD44. We find that the resistance to tensile stress for these hyaladherins correlates with the size of the HA-binding domain. The lowest mean rupture forces are observed for members of the type A subgroup (i.e., with the shortest HA-binding domains; TSG-6 and HARE). In contrast, the mechanical stability of the bond formed by aggrecan in complex with cartilage link protein (two members of the type C subgroup, i.e., with the longest HA-binding domains) and HA is equal or even superior to the high affinity streptavidin•biotin bond. Implications for the molecular mechanism of unbinding of HA•hyaladherin bonds under force are discussed, which underpin the mechanical properties of HA•hyaladherin complexes and HA-rich extracellular matrices.

INTRODUCTION

Hyaluronan (HA) is an abundant and vital element of the extracellular matrix in all vertebrates. It is a linear polymer with typical molecular weights on the order of 1 MDa, corresponding to contour lengths of several micrometers, and composed of repeated disaccharide units of glucuronic acid and N-acetylglucosamine, which are linked by alternating β-1,4 and β-1,3 glycosidic bonds. Despite having a regular structure—much simpler than the other (heterogeneously sulfated) members of the glycosaminoglycan family, such as heparan sulfate, chondroitin sulfate, and keratan sulfate—HA has a central role in regulating various pathological and physiological processes, such as inflammation, immune response, embryogenesis, tumor development, osteoarthritis, and atherosclerosis (1–4). The diverse biological functions of HA arise from its interactions with a wide range of proteins in the extracellular matrix and on the cell surface, collectively known as hyaladherins.

Submitted January 3, 2018, and accepted for publication May 7, 2018.
*Correspondence: r.richter@leeds.ac.uk
Editor: Jennifer Curtis.
https://doi.org/10.1016/j.bpj.2018.05.014
© 2018 Biophysical Society.

Hyaladherins bind to the flexible and large HA chains and promote their self-assembly into hydrogel-like multimolecular complexes that frequently undergo further dynamic remodeling (1,5). HA•protein interactions have a structural role in the extracellular space and thus are subjected to mechanical forces when matrices or tissues are deformed. For example, large supramolecular complexes made from HA and aggrecan, a proteoglycan with a molecular structure akin to that of a bottle brush, make a vital contribution to the integrity and biomechanical properties of cartilage that are crucial for joint function (6–8). In this scenario, the G1 domain on the N-terminus of aggrecan binds to HA, where this interaction is stabilized by cartilage link protein (LP), which simultaneously binds HA and aggrecan. Similarly, complexes of HA with versican, another proteoglycan with a bottle-brush-like structure, contribute to the elasticity of blood vessel walls, and mechanical strain has indeed been observed to modulate versican expression and organization by vascular smooth muscle cells (9). In these and other contexts, the protein tumor necrosis factor-stimulated gene 6 (TSG-6) is thought to promote the dynamic remodeling of HA-rich matrices under inflammatory conditions, e.g., by

cross-linking HA (1,10). Mechanical forces also play an important role in the engagement of HA with cell surface receptors. For example, interactions of HA with the receptor CD44 have been implicated in the recruitment of immune cells (11,12), stem cells, and cancer cells (13,14) from the blood circulation, where the HA•CD44 bonds formed between the luminal blood vessel walls and the circulating cell experience the shear stress of the blood flow. Moreover, the cellular uptake of HA via endocytosis is also likely to expose the bonds between HA and its receptors (such as the HA receptor for endocytosis, HARE, also called stabilin-2 (15)) to mechanical stress because of the packing constraints that are associated with the large size and flexibility of HA.

The above examples illustrate the functional relevance of HA•hyaladherin bond nanomechanics in various physiological and pathological contexts. However, only little is known about the resistance of HA•hyaladherin interactions to mechanical stress at the molecular level; some data are available for HA•CD44 (16–18), but the nanomechanical properties of bonds between HA and other hyaladherins have, to our knowledge, not yet been quantified.

About a dozen hyaladherins are currently known that belong to the so-called Link module superfamily (19,20) and selectively bind HA through one or two concatenated Link modules. Link modules are domains of ~100 amino acids with structural similarities to the C-type lectin domain (21,22). Based on the size of the HA-binding domain, the Link module superfamily has been discriminated into three subtypes (23). Type A has a single folded Link module (~90 amino acids), and TSG-6 and HARE belong to this subtype. HA-binding domains of type B are larger (~160 amino acids) and feature a Link module with extensions at the N- and C-terminals that are critical for structural and functional activity. CD44 is an example of type B hyaladherins (24). Type C has the largest HA-binding domains (~200 amino acids), which comprise two contiguous Link modules; aggrecan (25), versican (26), and LP all belong to this type.

The size of the HA-binding domain in the Link module superfamily broadly correlates with the minimal size of HA that is required for full binding activity. For example, a heptasaccharide (HA_7) is sufficient to fill the HA-binding site of TSG-6 from type A (22), whereas, a decasaccharide (HA_{10}) is typically required to reach close-to-maximal affinity for versican from type C (27). For CD44 from type B, an octasaccharide (HA_8) is required for close-to-maximal affinity (28). An interesting question is whether the mechanical strength of HA•hyaladherin bonds also correlates with the size of the HA-binding domain.

In this study, we analyze and compare the nanomechanical properties of a range of HA•hyaladherin complexes that covers all subtypes of the Link module superfamily and also includes a ternary HA•hyaladherin complex in addition to the binary complexes. Specifically, we use atomic force microscopy (AFM) based single-molecule force spectroscopy (SMFS) to quantify the response of individual molecular interactions to tensile forces. This force probe technique is now well established for the analysis of intra- and intermolecular forces (29,30) and is emerging for the probing of glycosaminoglycan-protein interactions (16,31,32). A prerequisite of AFM SMFS measurements is the proper immobilization of the molecules to be probed, in a way that permits the controlled application of the necessary tensile forces. We have recently reported a versatile AFM SMFS method to analyze HA•hyaladherin interactions (16). In this approach, we gave particular attention to the immobilization of HA. HA polymer chains were immobilized via one of their two ends (the so-called reducing end) to the sharp apex of an AFM tip, and this enabled both monovalent and multivalent interactions between a single HA chain and a hyaladherin-coated surface to be probed. Here, we apply this method and systematically quantify the resistance of individual HA•hyaladherin molecular interactions to tensile forces as a function of the hyaladherin type and loading rate.

As model protein constructs for our study (Fig. 1 *A*), we used the Link module of TSG-6 (TSG6_LM) and the extracellular domain (ECD) of HARE (HARE_ECD) to represent type A hyaladherins and the G1 domain of versican (VG1) to represent type C hyaladherins; we then compared the results with previously reported data (16) for the ECD of CD44 as a representative of type B hyaladherins. In addition, we used the G1 domain of aggrecan (AG1) in complex with cartilage LP (AG1•LP) to probe the mechanical stability of a ternary complex with HA. We show that the mechanical stability of individual bonds between HA and hyaladherins varies moderately but systematically with hyaladherin subtype: for any given loading rate, the mean bond rupture forces are lowest for type A hyaladherins and highest for type C hyaladherins. Moreover, we find that ternary AG1•LP•HA complexes are very strong and exceed the mechanical stability of streptavidin•biotin bonds.

MATERIALS AND METHODS

Proteins, HA, and buffer

Complexes of aggrecan G1 domain and cartilage LP (AG1•LP) were purified from bovine articular cartilage and biotinylated by N-hydroxysuccinimide-mediated labeling of surface amines, as described previously (33). Lyophilized AG1•LP was dissolved in working buffer to make a stock solution at 1 mg/mL protein concentration.

Recombinant human versican G1 domain (VG1) expressed in *Escherichia coli*, either without tags or biotinylated, were purchased from Antibody BCN (Barcelona, Spain) and delivered in phosphate-buffered saline (PBS) (pH 7.4) with 10% glycerol at a protein concentration of 0.25 mg/mL.

A fusion protein consisting of the human TSG-6 Link module (amino acids 18–111 of TSG-6; with a mutationally inactivated heparin-binding domain) at the C-terminal section and the Fc region of human IgG1 at

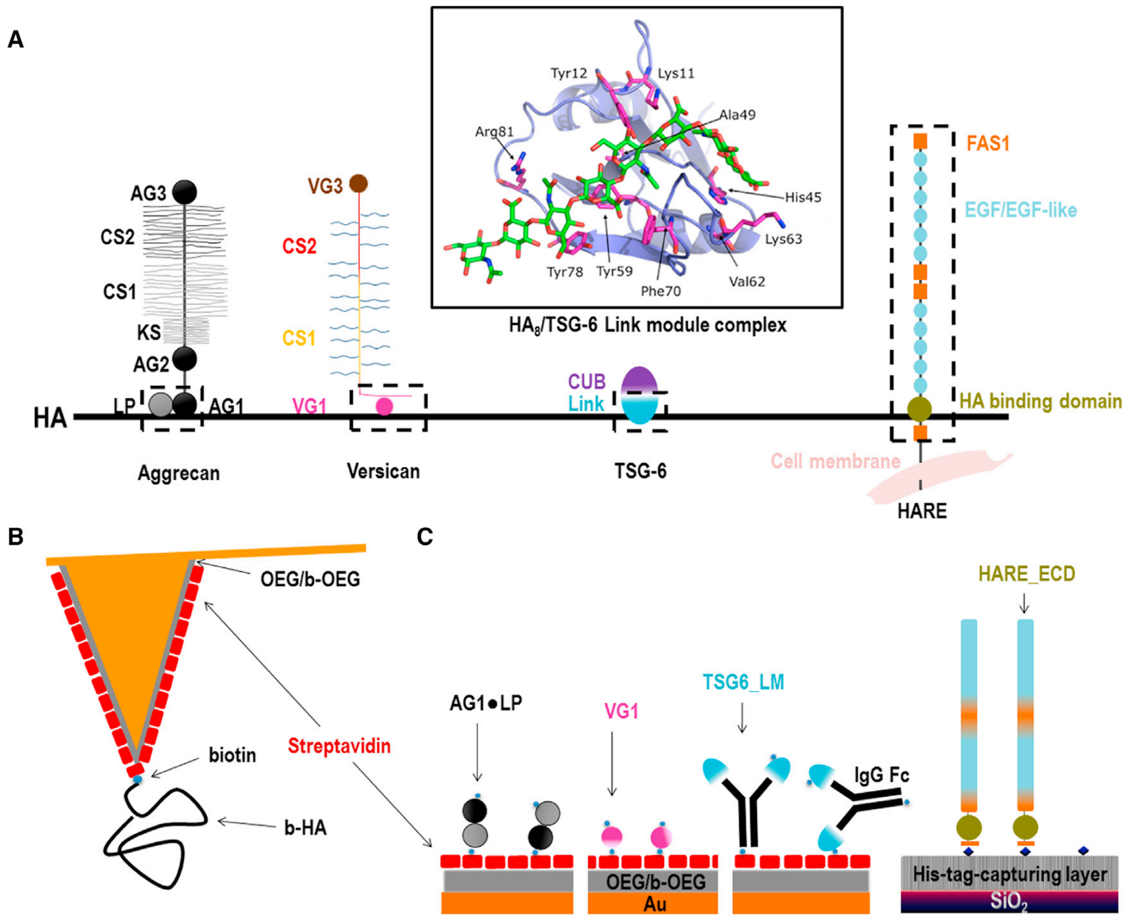

FIGURE 1 (*A*) Schematic representations (not to scale) of the protein constructs used and the hyaladherins from which they are derived. Dashed boxes contain the HA-binding regions that are used in this study. Schematics for aggrecan, versican, TSG-6, and HARE were adapted from (1,26,71,72), respectively. The inset shows a model of an HA octasaccharide (in *stick* representation) in complex with the TSG-6 Link module (in *ribbon* representation, with aminoacids that interact with HA indicated and in *stick* representation) taken from (22). (*B*) Schematic representation (not to scale) shows AFM tip functionalization with 2.1 μm (840 kDa) long end-biotinylated HA (b-HA). (*C*) Schematic representations show surface functionalization with hyaladherins, with the size of all proteins drawn roughly to scale. The TSG-6 link module is expected to be displayed as a dimer because of the dimeric IgG Fc region to which it is fused. CS, chondroitin sulfate; KS, keratan sulfate; LP, link protein; AG1/2/3, aggrecan G1/2/3 domain; VG1/3, versican G1/3 domain; CUB, CUB module; Link, Link module; EGF/EGF-like, epidermal growth factor (like) domains; FAS1, Fasciclin 1 domains; OEG, oligo(ethylene glycol); b-OEG, biotinylated OEG; IgG Fc, Fc domain of IgG; TSG_LM, TSG-6 link module fused to IgG Fc; HARE_ECD, HARE extracellular domain. To see this figure in color, go online.

the N-terminal section (TSG6_LM) was recombinantly expressed in Chinese hamster ovary S cells and purified as described elsewhere (34) and was either used as is or biotinylated by N-hydroxysuccinimide-mediated labeling of surface amines (34). We note that each TSG6_LM is expected to contain two TSG-6 Link modules because of the formation of disulfide-bonded dimers at the Fc region. The concentration of the TSG6_LM stock solutions was 0.25 mg/mL in PBS.

The ECD of the 190 kDa isoform of the HA receptor for endocytosis (HARE_ECD) in which the transmembrane and cytosolic domains at the C-terminus are replaced by a hexahistidine tag was constructed as described earlier (35). To purify the protein, conditioned medium (250 mL) from Flp-In HEK293 cells stably expressing the recombinant HARE_ECD was incubated for 18 h with 1 mL of packed resin conjugated with monoclonal antibody 30 (15) overnight at 4°C under slow rotation. The mixture was poured through an empty column (20 mL PolyPrep column; BioRad, Hercules, CA) to collect the resin, which was then washed with PBS (147 mM NaCl, 20 mM Na_2HPO_4 (pH 7.2)), and HARE_ECD was eluted from the resin with 100 mM glycine (pH 3.0) and immediately neutralized in excess unbuffered 1.0 M Tris base. The protein was concentrated (Vivaspin Turbo 4 with 100 kDa molecular weight cutoff; Sartorius, Bohemia, NY) and buffer exchanged in PBS at a concentration of 0.3 mg/mL. Purity of the preparation was assessed by sodium dodecyl sulfate polyacrylamide gel electrophoresis followed by silver staining the gel, and concentration was evaluated with the bicinchoninic acid assay.

Lyophilized streptavidin (Sigma Aldrich, St. Louis, MO) was dissolved in ultrapure water (resistance 18.2 MΩ · cm at 25°C; Barnstead Nanopure Diamond, Thermo Fisher Scientific, Waltham, MA) at 1 mg/mL.

Lyophilized HA polymer with well-defined molecular masses (select HA) was obtained from Hyalose (Oklahoma City, OK). HA with a biotin at its reducing end (b-HA) had a molecular mass of 840 ± 60 kDa, and unmodified HA had a molecular mass of either 250 ± 12 kDa (for binding assays) or 58 ± 3 kDa (for blocking assays). HA was dissolved and gently shaken for 2 h in ultrapure water to provide a stock of 1 mg/mL. Stock solutions of all proteins and HA were aliquoted and stored at −20°C. Thawed aliquots of proteins were used within a few days, and thawed aliquots of HA were used within a few weeks.

A working buffer consisting of 10 mM HEPES and 150 mM NaCl at pH 7.4 was used to dilute all protein and HA stocks to working concentrations

and for all quartz crystal microbalance with dissipation monitoring (QCM-D) and SMFS measurements performed throughout this study. For experiments concerning TSG6_LM, the working buffer was supplemented with 2 mM $CaCl_2$.

Substrates

QCM-D sensors with gold coating (QSX301; Biolin Scientific, Västra Frölunda, Sweden) were used as received or after recoating with an additional 100 nm gold film. QCM-D sensors with a His-tag-capturing coating (QSX340; Biolin Scientific) were used as received or after regeneration with solutions of imidazole in ultrapure water (25 min at 500 mM) and, subsequently, $CuSO_4$ in working buffer (15 min at 5 mM). Gold-coated AFM cantilevers with nominal spring constants of 30 or 6 pN/nm (Biolevers) and 60 pN/nm (NPG-10) were purchased from Bruker AFM Probes (Santa Barbara, CA).

Functionalization of gold surfaces with a biotin-displaying oligo(ethylene glycol) monolayer

Functional oligo(ethylene glycols) (OEGs) were purchased from Polypure (Oslo, Norway); one was made of seven ethylene glycol units with a hydroxyl group on one end and a thiol on the other (OEG thiol), and the other contained 10 ethylene glycol units with a biotin on one end and a thiol on the other (b-OEG thiol). Gold-coated planar substrates or AFM cantilevers were exposed to ultraviolet/ozone for 30 min and then immersed overnight at 4°C in an ethanolic solution (purity 99.9%; Scharlab S.L., Barcelona, Spain) of OEG thiol and b-OEG thiol at a total concentration of 1 mM and a molar ratio of 99:1. Before use, the functionalized substrates were rinsed with ethanol and blowdried with N_2 gas. The biotinylated thiol-OEG monolayer is inert to the nonspecific binding of proteins but permits the stable and specific binding of streptavidin via interactions with biotins (16,36), where this interaction is multivalent with typically two or three biotin•streptavidin bonds per streptavidin molecule (37). It was prepared ex situ before QCM-D or SMFS measurements on biotin-tagged proteins and HA.

Anchoring HA to the AFM tip

HA polymers (840 $\pm$ 60 kDa; contour length 2.10 $\pm$ 0.15 μm) were attached to gold-coated AFM cantilevers through a single biotin tag at the reducing end to a streptavidin monolayer on a biotinylated thiol-OEG monolayer (Fig. 1 *B*). Details of the method have recently been reported (16). Briefly, the cantilevers with a biotinylated thiol-OEG monolayer were first incubated in a streptavidin solution with incubation conditions (20 min at 20 μg/mL) leading to the formation of a dense protein monolayer in which each streptavidin molecule is attached to multiple biotins (37). The cantilevers were then immersed in a solution of b-HA at conditions (6 min at 2 μg/mL) that produce a low HA surface coverage. Specifically, we estimate a root mean-square distance between anchor points of the AFM tip to be 76 nm (16,38). Considering the large radius of gyration of the HA polymer (radius of gyration ~75 nm (39)) and the small radius of the AFM tip apex (30 nm), it can be expected that only one or at most a few HA chains can contact the surface simultaneously, thus facilitating the probing of individual HA•hyaladherin interactions.

QCM-D

QCM-D measures the changes in resonance frequency, Δf, and dissipation, ΔD, of a sensor crystal upon molecular adsorption on its surface. The QCM-D response is sensitive to the areal mass density (including hydrodynamically coupled water) and the mechanical properties of the surface-bound layer. To a first approximation, a decrease in frequency (Δf) corresponds to increased mass, whereas a low (high) response in dissipation (ΔD) corresponds to a rigid (soft) film.

QCM-D measurements were carried out with a Q-Sense E4 system equipped with Flow Modules (Biolin Scientific) with flow rates of 5–20 μL/min at a working temperature of 23°C. Before all experiments, the walls of chambers and tubings were passivated by exposure to 10 mg/mL bovine serum albumin for 20 min followed by rinsing in ultrapure water and blowdrying with N_2 gas.

Δf and ΔD were collected at six overtones (i = 3, 5, 7, 9, 11, and 13). Changes in dissipation, ΔD, and normalized frequencies, $\Delta f = \Delta f_i/i$, for $i = 3$ are presented. Any other overtone would have provided similar information. For the sake of clarity, we subtracted contributions of the sample solution (because of changes in the viscosity and/or density compared to working buffer) from the displayed QCM-D responses; this was necessary for the VG1 incubation step (Fig. 2 *B*; offsets were $\Delta f = 1.2 \pm 0.3$ Hz and $\Delta D = -0.6 \pm 0.1 \times 10^{-6}$; cf. Fig. S1, *B* and *C*) and the HARE_ECD incubation step (Fig. 2 *D*; offset was $\Delta D = -0.4 \pm 0.1 \times 10^{-6}$; cf. Fig. S1 *E*). All experiments were carried out in duplicate; numbers in the text represent the mean $\pm$ variations around the mean.

For dense monolayers of globular proteins, the film thickness was estimated from $d = -C/\rho \times \Delta f$, where the density $\rho = 1.2$ g/cm^3 represents the protein film density to within an error of less than 20% and $C = 18.1$ ng $\cdot$ cm^{-2} $\cdot$ Hz^{-1} represents the sensor's mass sensitivity constant (40).

SMFS

AFM SMFS experiments were carried out on a NanoWizard II system (JPK, Berlin, Germany) in working buffer at ambient conditions using gold-coated cantilevers: OBL with a nominal spring constant of 6 pN/nm and NPG with a nominal spring constant of 60 pN/nm (both from Bruker AFM Probes). Cantilever spring constants were determined by the thermal noise method (41) and found to be within 10% of the nominal values provided by the manufacturer. Force curves were registered at selected retract speeds with a maximal applied load of 600 pN and a minimal surface dwell time (i.e., 0 ms). For a given set of AFM probes, surface and interaction settings between several 100 and a few 1000 force curves were collected (Table S1). All experiments were performed at least twice with different yet identically prepared AFM probes and surfaces. Moderate variations in the noise of force curves across measurements are due to variations in the AFM probes.

Force curves were analyzed with JPK data processing software. For quantitative analysis of the stretching of individual HA chains and to extract bond rupture forces, force-separation curves were fitted with the worm-like chain (WLC) model (42) with both persistence length and contour length as adjustable parameters. Only rupture events appearing at tip-sample distances larger than 200 nm were considered for further analysis to avoid bias by nonspecific tip-sample interactions. Instantaneous loading rates r were calculated from the effective spring constant k_{eff}, corresponding to the slope of the WLC fit close to the rupture (Fig. S2 *A*) and the retract velocity v as $r = k_{\text{eff}} v$. These loading rates agreed (to within a few percentages) with the expectations according to the theoretical expressions for the force-dependent loading rate established by Dudko et al. (43). Mean rupture forces were determined through Gaussian fits on force histograms. Whereas the Bell-Evans model relies on the analysis of the most likely rupture force (44), we found the mean rupture force to be a good approximation considering the SD and shape of the experimental force histograms. OriginPro software (OriginLab, Northampton, MA) was employed for nonlinear regression analysis to extract the effective kinetic parameters (k_{off} and x_β) from the data of mean rupture force versus instantaneous loading rate using the Bell-Evans model (29,45). In this analysis, the SE of the mean rupture force was considered to determine the confidence interval for the kinetic parameters.

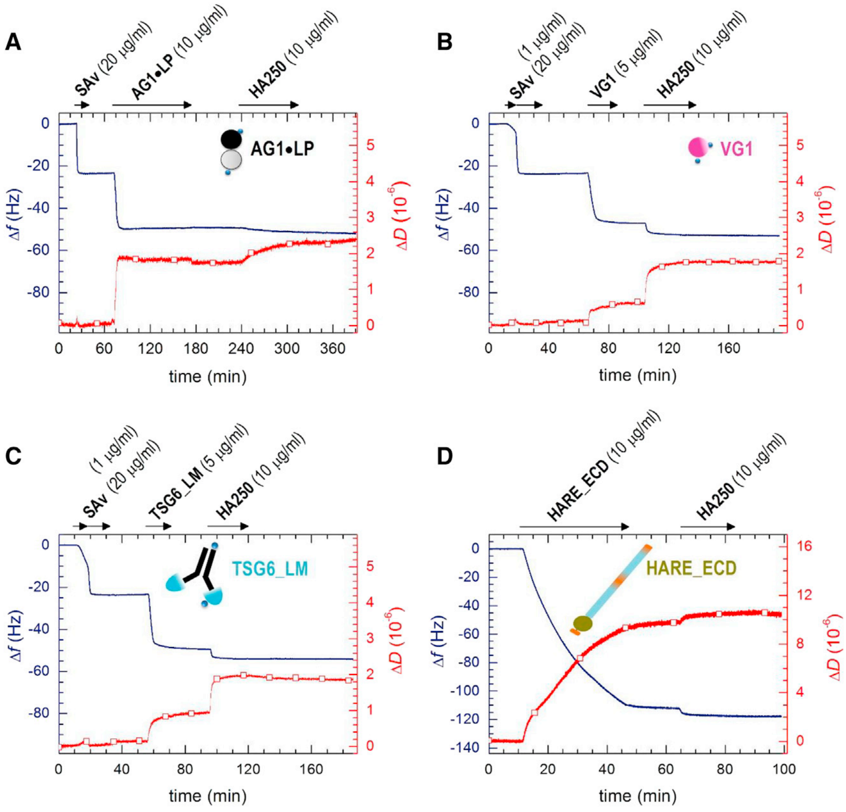

FIGURE 2 QCM-D analysis of hyaladherin immobilization and HA-binding. Frequency shifts, Δf, are shown as lines, and dissipation shifts, ΔD, are shown as red lines with open squares. The start and duration of incubation with different samples are indicated by arrows on top of the graphs; remaining times represent washing steps with working buffer. QCM-D responses indicate formation of a stable and dense streptavidin monolayer (on sensors with a biotin-displaying thiol-oligo(ethylene glycol) monolayer on a gold surface), followed by the formation of stable monolayers of AG1•LP (*A*), VG1 (*B*), and TSG6_LM (*C*); the QCM-D response in (*D*) demonstrates the formation of a stable HARE_ECD monolayer (on a sensor with a His-tag-capturing surface). HA-binding can be observed on all hyaladherins, although binding is slower and less pronounced in the case of AG1•LP (*A*), indicating that the surface density of active HA-binding sites is low for this hyaladherin. To see this figure in color, go online.

RESULTS

Analysis of hyaladherin immobilization and HA binding

Before embarking on the analysis of the mechanical properties of individual HA•hyaladherin interactions, we verified that all hyaladherins can be immobilized stably and retain their ability to bind HA when immobilized. QCM-D was used to monitor the assembly of the protein films and HA binding; the measurement of two distinct parameters—the QCM-D sensor's shifts in resonance frequency Δf and dissipation ΔD—provides simultaneous and time-resolved information about binding processes and about the morphology and mechanical properties of the biomolecular films. The design of the immobilization strategies is schematically shown in Fig. 1 *C*. AG1•LP, VG1, and TSG6_LM were immobilized via their biotin tags on dense monolayers of streptavidin formed on gold-supported biotin-displaying monolayers of thiol-terminated OEG on gold surfaces. HARE_ECD was immobilized via its His_6 tag to a His-tag-capturing surface displaying a Cu^{2+} chelate. The location of the histidine tag at the C-terminus endows HARE_ECD with a well-defined attachment point equivalent to that of the full-length protein in the cell membrane. In contrast, biotins on AG1•LP, VG1, and TSG6_LM are likely to be present on multiple surface amines, and these proteins may therefore attach to the surface in multiple distinct orientations via one or several biotins.

The QCM-D responses upon incubation with streptavidin (20 μg/mL, Fig. 2, *A–C*) are in good agreement with earlier studies and indicate the formation of a dense and stable streptavidin monolayer of ~4 nm thickness that serves as a "molecular breadboard" (16,36,46) to anchor the desired biotinylated hyaladherins.

The QCM-D responses upon incubation with AG1•LP (10 μg/mL) indicate stable immobilization of the protein in a monolayer of 5 nm thickness (Fig. 2 *A*). HA polymer (250 kDa), incubated at 10 μg/mL, bound stably to AG1•LP (Fig. 2 *A*). Control experiments confirmed that AG1•LP binding to the streptavidin monolayer is largely blocked when streptavidin is saturated with free biotin before AG1•LP exposure and that HA binding is fully specific for AG1•LP that is immobilized through biotins (Fig. S1 *A*). It is notable though that HA binding is relatively slow and that the concomitant shifts in frequency (-1.7 ± 0.9 Hz) and dissipation ($0.5 \pm 0.2 \times 10^{-6}$) are relatively small (vide infra). The latter suggests that only a small fraction of the immobilized AG1•LP is active. Presumably, the random orientation enables binding only to a subset of suitably oriented complexes.

The QCM-D responses for VG1 also indicate stable and specific immobilization of the protein in the form of a monolayer (Figs. 2 *B* and S1, *B* and *C*). The frequency shift

at equilibrium after incubation with VG1 (5 μg/mL) and washing in working buffer ($\Delta f = -23 \pm 1$ Hz) corresponds to a film thickness of ~4 nm, which is consistent with the size of a protein globule of the molecular mass of VG1 (36.7 kDa). VG1 without biotin tags did not bind (Fig. S1 *B*), confirming the specific binding of VG1 to streptavidin through biotin. HA binding to the VG1 monolayers was rapid and strong ($\Delta f = -5.7 \pm 0.5$ Hz, $\Delta D = 1.2 \pm 0.1 \times 10^{-6}$), confirming the activity of the immobilized protein.

The QCM-D responses upon exposure of 5 μg/mL of biotinylated TSG6_LM to a streptavidin monolayer again clearly demonstrate stable binding in the form of a monolayer (Fig. 2 *C*). The shift in frequency of -26 ± 1 Hz corresponds to an effective film thickness of ~5 nm. This is smaller than the longest extension of the Fc region (7 nm (47)) plus the size of the TSG-6 Link module (3 nm (36)) and suggests that the dimeric TSG6_LM molecules would lie mostly flat on the streptavidin monolayer. However, HA binding to TSG6_LM was rapid and strong; shifts in frequency and dissipation of -4.6 ± 0.7 Hz and $0.9 \pm 0.1 \times 10^{-6}$, respectively, were comparable to those observed for VG1, indicating that TSG6_LM retains good HA binding in the flat orientation. Like for VG1, TSG6_LM lacking biotin tags did not bind to streptavidin (Fig. S1 *D*), confirming specific immobilization through biotin.

Fig. 2 *D* demonstrates strong and stable binding of HARE_ECD to the His-tag-capturing surface. The frequency shift for this protein was much larger than for any of the other studied hyaladherin constructs. After 30 min of HARE_ECD incubation at 10 μg/mL, $\Delta f = -110 \pm 4$ Hz was attained, equivalent to ~17 nm in film thickness. That the protein could be completely eluted with imidazole (Fig. S1 *E*) indicates specific surface attachment through its C-terminal His_6 tag. The QCM-D data thus are fully consistent with a binding scenario in which the HA-binding domain, which is close to the C-terminus in HARE_ECD (Fig. 1), is located in the vicinity of the surface, whereas the remaining large multidomain region with the N-terminus is allowed to dynamically flex into the solution phase. We have here not attempted to drive the HARE_ECD film formation to saturation, as this process can take a long time; as the film becomes denser, an entropic barrier is generated because of the extended shape and flexibility of HARE_ECD that gradually reduces the protein-binding rate. HA polymer of 250 kDa incubated at 10 μg/mL bound stably to immobilized HARE_ECD ($\Delta f = -5.4 \pm 0.1$ Hz, $\Delta D = 1.0 \pm 0.1 \times 10^{-6}$). The initial rate of HA binding to HARE_ECD and the associated magnitude of the QCM-D response were similar to VG1 and TSG6_LM (Fig. 2), indicating proper access of the HA-binding domain to HA.

Taken together, we conclude from the QCM-D data (Figs. 2 and S1) that all here-studied hyaladherin constructs can be immobilized stably and specifically and that they retain their ability to bind HA when immobilized, to a great extent for VG1, TSG6_LM, and HARE_ECD and to a lesser extent for AG1•LP.

Force spectroscopy of single HA•hyaladherin bonds

We then studied and compared the dynamics of the four HA•hyaladherin interactions under force by SMFS. To this end, HA polymers were grafted at low density to sharp AFM tips such that only one or at most a few HA chains can contact the surface simultaneously (Fig. 1 *B*), thus facilitating the probing of single HA•hyaladherin interactions (16). HA does not self-associate under the conditions of our assay (48), and its extension upon tensile force thus is dominated by the elastic stretching of flexible polymer chains. Hyaladherins were immobilized on planar surfaces using the methods established by QCM-D but with conditions adjusted to achieve predominantly single-binding and unbinding events. Where needed, the hyaladherin surface densities could be easily tuned by adjusting the protein concentration or incubation time. For AG1•LP, the HA-binding activity was relatively low (cf. Fig. 2 *A*), and no changes to the immobilization conditions were effectively required. For VG1 and TSG6_LM, substantial reductions in the incubation concentration (from 5 to 1 μg/mL) and the incubation time (from 10 min to 5 s) were required, which was consistent with the fast and strong binding of HA to these proteins (cf. Fig. 2, *B* and *C*). Considering that mass transport limits the binding of biotin to streptavidin, it can be estimated that the root mean-square distance between immobilized proteins with such a brief incubation lies roughly between 50 and 100 nm (49), a distance that is comparable to the radius of gyration of the HA polymer used here (75 nm (39)). Like for AG1•LP, immobilization conditions for HARE_ECD were also not changed, implying much denser protein coatings. A possible explanation for the lower activity of HARE_ECD over VG1 and TSG6_LM in the SMFS assays is that the large N-terminus delays access of HA to the HA-binding domain of HARE. Although QCM-D (Fig. 2) did not show any reduced HA-binding rate for HARE_ECD compared to VG1 and TSG6_LM, this may well be because of mass transport limiting the binding in all three cases.

Representative force-separation curves, obtained by bringing HA-modified AFM tips into contact with hyaladherin-coated surfaces, are shown in Fig. 3. These curves show the typical features expected for the elastic stretching of an HA chain followed by a rupture event.

Force curves were acquired over a range of retract velocities, with between many hundreds and a few thousand force curves per velocity for each of the hyaladherins (Table S1), and from their analysis, we can conclude that interactions between a single HA chain and a single protein/receptor are being probed in most cases. First, the overall probability (including all tested retract velocities) of a single specific

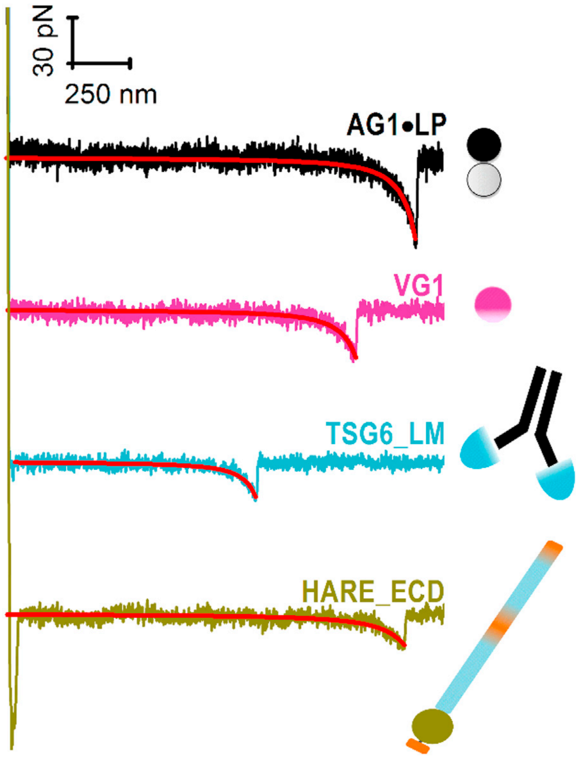

FIGURE 3 Representative examples of force-distance curves upon separation of the HA-coated AFM tip from hyaladherin-coated surfaces (as indicated) showing a single unbinding event. The maximal compressive load upon approach was 600 pN, and force curves were recorded at a retract speed of 1000 nm/s; red lines are fits to the worm-like chain (WLC) model. To see this figure in color, go online.

rupture event to occur was between 6 and 19%, whereas the majority of force curves showed no specific rupture events at all, and only a small fraction (<2%) displayed two or more rupture events (cf. Fig. S2, *empty bars* in (*B*)–(*E*), showing data for a selected retract velocity of 2000 nm/s). This validates that the stochastic rupture of mostly single bonds has been probed. Second, all force curves showing a single rupture event fell onto a single master curve when properly normalized for variations in the locus of hyaladherin binding along the HA homopolymer chain (Fig. S3) (50). Third, nonlinear regression analysis of the force curves with the WLC model revealed a velocity-independent persistence length of $L_p = 4.2 \pm 0.2$ nm (Fig. S4). The magnitude of L_p agrees with values previously obtained by us (4.1 $\pm$ 0.4 nm (16)) and others (4.4 $\pm$ 1.2 nm (51)) from single-molecule HA stretching experiments at comparable solution properties, indicating that a single HA chain is being stretched. Finally, the contour length L_c between the anchor point of HA and the hyaladherin binding locus varied broadly between measurements, and the maximal observed value was comparable to the total contour length of the employed HA chains (2.1 μm; Fig. S5). This confirms that hyaladherins can bind at any position along the HA chain, as expected given that HA is a linear homopolymer. Moreover, to ascertain that the specific binding of HA to hyaladherins is being probed in the force spectroscopy assays, competition assays with shorter HA polymers (58 kDa; 10 μg/mL) in the solution phase were performed. Under these conditions, the probability to detect HA stretching and bond breakage was indeed reduced substantially (Fig. S2, *solid bars* in (*B*)–(*E*)).

A detailed statistical analysis of the rupture forces obtained from all force curves showing a single specific rupture event is presented in Fig. 4. All histograms of rupture forces showed unimodal distributions that could be approximated reasonably well by Gaussians. The resulting mean rupture forces likely deviate only little from the most probable rupture forces (because the histograms are quite symmetric) and are displayed as a function of loading rate in Fig. 5. For completeness, we include equivalent data for the ECD of CD44 (CD44_ECD), which we have previously reported (16). A main result of this analysis is that the mean rupture forces over the range of loading rates probed are the lowest for the type A hyaladherins TSG6_LM (19–32 pN) and HARE_ECD (17–30 pN), intermediate for the type B hyaladherin CD44_ECD (31–45 pN) and the type C hyaladherin VG1 (26–48 pN), and highest for the complex of the two type C hyaladherins AG1•LP (34–78 pN).

For comparison, the dynamic force spectra in Fig. 5 also show results for the rupture of individual streptavidin•biotin bonds (*gray squares*) that we have previously reported for an experimental setup in which streptavidin is immobilized in the same way as done in this study (16) (Fig. S6). Such a comparison is important because streptavidin•biotin bonds were used to anchor HA and hyaladherins in our assays. Using CD44_ECD, we have previously shown that the anchorage via a biotin tag to the streptavidin monolayer is strong enough such that its breakage is rare compared to the breakage of CD44_ECD•HA bonds (16). The rupture forces for TSG6_LM and VG1 are clearly below the forces required for breaking streptavidin•biotin bonds. Likewise, the rupture forces for HARE_ECD are much lower than those previously reported for chelates of penta- or hexahistidines (52,53). This implies that a breakage of anchors (biotin or His tags) is rare in these cases, and we can therefore conclude that the genuine interactions of TSG6_LM, HARE_ECD, and VG1 with HA have been quantified. For AG1•LP, on the other hand, the measured rupture forces are virtually identical to those of streptavidin•biotin bonds. This indicates that the mechanical stability of AG1•LP bonds is comparable to or even higher than that of streptavidin•biotin bonds. As a consequence, the exact magnitude of AG1•LP•HA rupture forces cannot be quantified with our setup, and our results instead represent a lower estimate. The complete overlap of the two data sets in Fig. 5 *E* also implies that the additional biotin•streptavidin bonds that are involved in load bearing in the HA•hyaladherin rupture assays do not reduce the mechanical stability appreciably

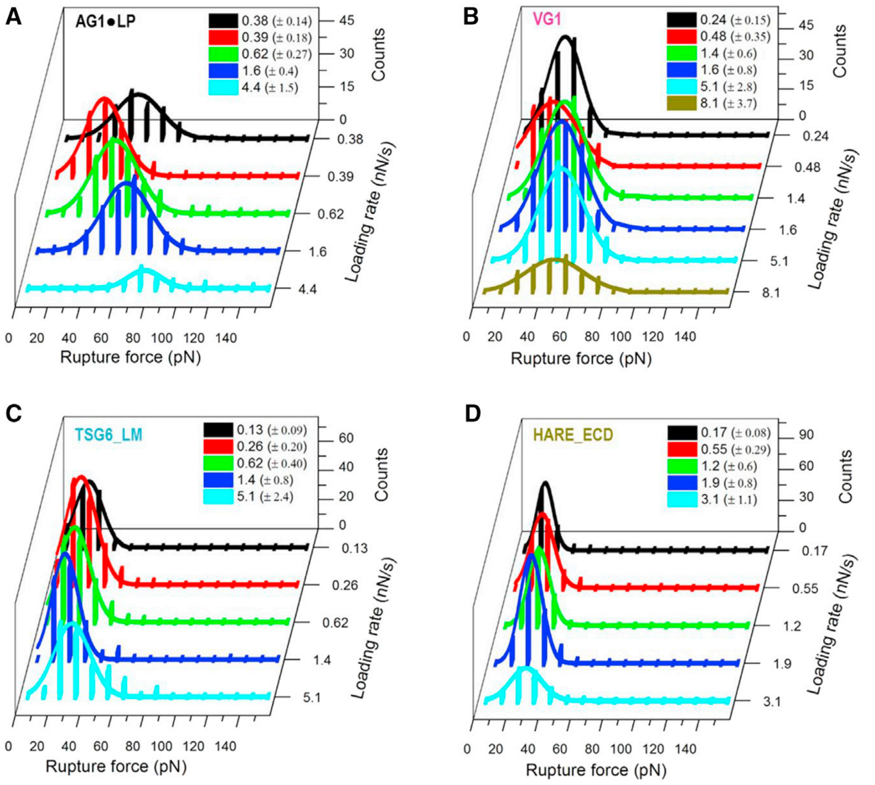

FIGURE 4 Rupture force histograms for various instantaneous loading rates (listed with grayscale/color codes as mean ± SD) for the interaction of HA with AG1•LP (*A*), VG1 (*B*), TSG6_LM (*C*), and HARE_ECD (*D*). Solid lines represent Gaussian fits. To see this figure in color, go online.

compared to the control measurement (Fig. S6), in which fewer biotin•streptavidin bonds are connected in series (54). Most likely, this is because the anchorages of streptavidin to the biotinylated OEG monolayers (Fig. 1, *B* and *C*) and possibly also of the biotinylated hyaladherins to streptavidin are multivalent and thus very stable such that effectively only a small number (one or two) of more fragile single biotin•streptavidin connections are present in both assays.

All plots in Fig. 5 show a roughly linear dependence of the mean (and to a good approximation the most probable) rupture force on the logarithm of the instantaneous loading rate. This is in line with the predictions of the Bell-Evans model, i.e., $F = (k_B T/x_\beta)\ln(r x_\beta / k_{off} k_B T)$, where $k_B T$ is the thermal energy, x_β is the width of the energy barrier, and k_{off} is the unbinding rate constant in the absence of an external load. A fit of the data sets with this model provides k_{off} and x_β, and values are provided in Fig. 5 for the corresponding hyaladherins.

DISCUSSION

We have undertaken a systematic analysis of the nanomechanical properties of HA•hyaladherin bonds for hyaladherins that cover all three subtypes of the Link module superfamily. The data for four binary complexes and one ternary HA•hyaladherin complex show a good correlation of the mechanical strength with the size of the HA-binding site (Fig. 5; Table 1). At the molecular level, this suggests that the contact between HA and the binding surface on the hyaladherin is not released gradually, in a zipper like fashion (akin to the peeling of tape, where the elementary connections that constitute a bond break in series and the rupture force are independent of the length of the binding interface; illustrated in Fig. 6, *right*). Instead, it appears more appropriate to picture the breakage as a scenario in which all elementary connections act in parallel and break simultaneously (Fig. 6, *left*). The relatively high persistence length of HA ($L_p \approx 4$ nm, corresponding to the contour length of four disaccharides) would be consistent with such a scenario, i.e., the local stiffness facilitates the distribution of force across a relatively long stretch of the HA chain.

We have already pointed out that AG1•LP, VG1, and TSG6_LM are likely to be immobilized in multiple distinct orientations because their biotinylation is not site specific. We were initially concerned that this may impact the direction of tensile force applied to the bond. However, each histogram of rupture forces showed only one significant peak (Fig. 4), suggesting that a single type of interaction has been probed irrespective of the hyaladherin type and loading rate. This would imply that, at least for VG1 and TSG6_LM, the mode of anchorage does not affect the bond mechanics appreciably. Tailored protein constructs with anchoring groups at defined positions may in the future enable us to dissect how the pulling geometry affects the resistance of

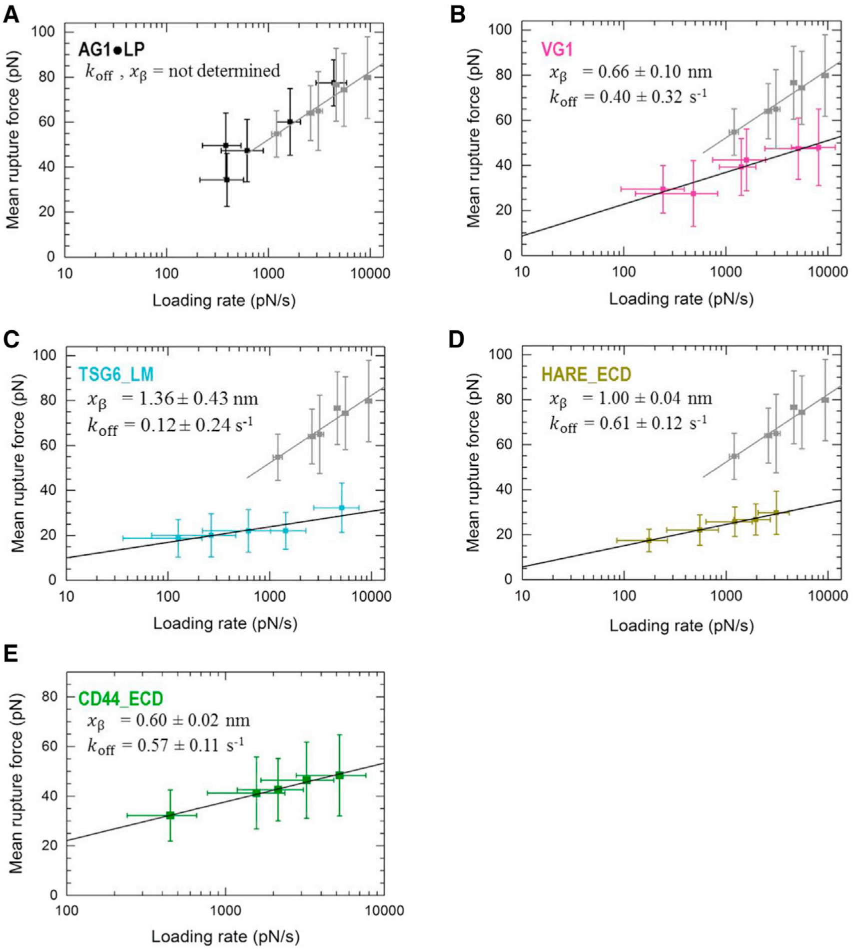

FIGURE 5 Dynamic force plots for the four hyaladherins studied here (*A–D*; obtained from Fig. 4) and for CD44_ECD (*E*; Fig. 4 from (16)). Mean rupture forces are presented as mean ± SD, and black lines are fits with the Bell-Evans model (resulting kinetic parameters are indicated; data for AG1•LP were not fitted, as here the streptavidin•biotin anchor is the weakest link). Dynamic force spectra for the rupture of streptavidin•biotin bonds are shown (in *gray*) for comparison (from (16); see also Fig. S6). To see this figure in color, go online.

HA•hyaladherin bonds, akin to recent work on streptavidin•biotin bonds (55).

It may here also be recalled that TSG6_LM contains two TSG-6 link modules because of their fusion to the dimeric Fc region (Fig. 1 *C*). This raises the question of whether the measured rupture forces (Figs. 4 *C* and 5 *C*) could be representative of the bond of a single HA chain with two, rather than one, TSG-6 link modules. We consider this possibility unlikely; the attachment to the Fc region imposes an antiparallel arrangement of the two link modules, and it is thus difficult for a single HA strand to form a continuous binding interface with the two modules. It is still possible that the long HA chain loops back in the antiparallel direction and then binds to both TSG-6 link modules on the same dimeric fusion molecule. However, this would likely result in two consecutive rupture events when the tensile force is applied along the HA chain, which is not supported by our experiments (Fig. S2 *D*).

The situation is different for AG1•LP, where the HA•AG1•LP complex was mechanically at least as strong

TABLE 1 Unbinding Forces at a Selected Instantaneous Loading Rate

Type	Hyaladherin	HA-Binding Domain Size (Amino Acids)	HA Minimal Binding Unit (Disaccharides)[a]	Bond Probed	Mean Rupture Force, *F* (pN)
A	TSG-6	~100[b]	4	TSG6_LM•HA	24
	HARE	93[c]	3	HARE_ECD•HA	25
B	CD44	~160[b]	4	CD44_ECD•HA	34[d]
C	VG1	~200[b]	5	VG1•HA	37
	AG1•LP	~200 + 200[b]	5 + 5	AG1•LP•HA	>52
Reference	–	–	–	streptavidin •biotin	52[d]

[a]Rounded to full disaccharides, the HA-binding site may be slightly shorter, e.g., in TSG-6 (22).
[b]Data were taken from (23).
[c]Link domain spanning from HARE Gly2198 to Tyr2290.
[d]Data were taken from (16).

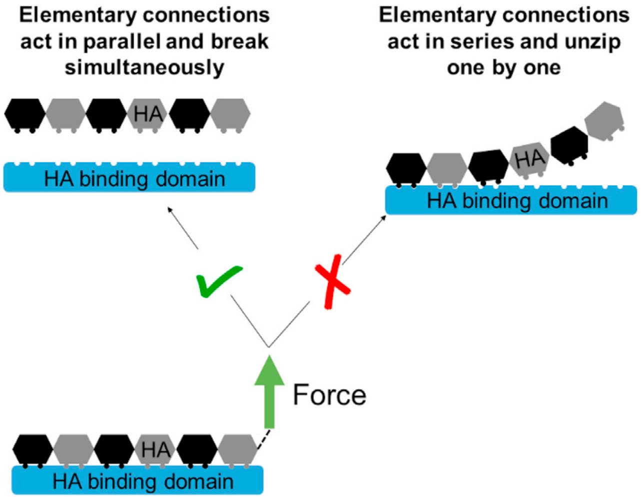

FIGURE 6 Schematic illustration of possible scenarios for breaking HA•hyaladherin bonds. The correlation of rupture force with bond length suggests that the elementary connections that constitute a bond act in parallel and break simultaneously, rather than in a zipper-like fashion. To see this figure in color, go online.

and most likely even stronger than the streptavidin•biotin anchor for the protein and HA. Although a different anchor design will be required to quantify the mechanical strength of the HA•AG1•LP complex in the future, our study already demonstrates that this ternary complex is substantially stronger than all binary HA•hyaladherin complexes tested and that it rivals one of the highest affinity noncovalent bonds in mechanical strength over the full range of loading rates tested. Our finding also has implications for the interpretation of an earlier study, in which the HA•AG1•LP complex was probed with optical tweezers using streptavidin-coated microbeads with biotinylated AG1•LP. Liu et al. (56) probed bond rupture at a single pulling speed and reported a rupture force of 40 $\pm$ 11 pN; the instantaneous loading rate was not provided in this study, but from the representative force curve shown in Fig. 1 of (56), we can estimate that it is on the order of 100 pN/s. At this loading rate, we also found rupture forces on the order of 40 pN (Fig. 5 *A*). The consistency with our data suggests that Liu et al. may unintentionally have probed streptavidin•biotin bonds instead of HA•AG1•LP bonds.

Over the range of loading rates covered in our experiments, the mechanical response of all tested HA•hyaladherin interactions was broadly consistent with the predictions of the Bell-Evans model; that is, bond rupture is adequately described by conventional unbinding across a single barrier, and the bond lifetime is predicted to decrease with increasing force ("slip bond"). In contrast, several groups have proposed that HA and CD44 may form bonds that have the unusual property to strengthen over a range of forces ("catch bond"). This hypothesis is based on the results of steered molecular dynamics simulations (18) and of experiments that probed the overall stability of large sets of bond acting together (18,57). Future experiments that probe a wider range of loading rates or directly measure the lifetime of individual HA•CD44 interactions at defined constant forces will be interesting to settle this question and to explore if other HA•hyaladherin interactions show an unusual dependence of their lifetime on force. Of particular interest will be the range of small forces (<20 pN) and loading rates (<100 pN/s), which was hardly accessible with our force ramp setup.

The trends in bond mechanical strengths reported in this article broadly correlate with the biological functions of the probed HA•hyaladherin complexes. The HA•AG1•LP is a core component of cartilage, which has a very slow turnover rate partly because of the high bond strength within this complex (27,58,59). Newly synthesized AG1 is highly modified in the endoplasmic reticulum of chondrocytes and is secreted for further processing of the core protein in the extracellular space for maturation of the G1 domain containing the HA-binding sites to form stable interactions with both LP and HA (60,61). Physiologically, one HA strand will host multiple AG1•LP complexes that are incorporated into cartilage, and the resulting complex has a half-life of 24 years (62). High bond strength and stability of the HA•AG1•LP local site is crucial for the longevity of cartilage tissue. This is in contrast to HARE, a scavenger receptor located within the solid-liquid interfaces of multiple tissues including the liver, spleen, and lymph node (63), which have high affinity for HA but must also release the HA within the endosomes and traffic back to the cell surface; thus, a lower bond strength would be advantageous (64). It is estimated that HARE recycles from the cell surface to recycling endosomes and back to the surface in less than 15 min, which strengthens the case for a weaker interaction with HA (65). The Link domains of HARE and TSG-6 have the highest level of sequence identity among any of the hyaladherins (66) and, correlating with this, the same order of mechanical HA-protein binding strength. Like HARE, TSG-6 is known to interact dynamically with HA in a pH-dependent manner, and as a soluble extracellular matrix protein, it supports the remodeling of HA-rich matrices within healthy and inflammatory tissues (36). What is most important for both HARE and TSG-6 is high specificity for the HA polymer but low bond strength between the protein and ligand because high turnover (i.e., release of cargo) is important for the functional roles of both proteins. The binding strength for HA•CD44 lies between HARE/TSG-6 and the HA•AG1•LP complex. CD44 is a cell surface receptor that interacts with the actin cytoskeletal system in quasi-stable structures and does not recycle through the endolysosomal system nearly as frequently as HARE (67). CD44 and its variants are implicated in probing the extracellular matrix and serve as tethers for cellular movement in a variety of cell types (e.g., immune cells and cancer cells), including under the shear stress of blood flow (11–14). Aside from its role in

attachment, a stable HA•CD44 interaction along with other accessory proteins promotes cellular signaling events that result in cell proliferation, dedifferentiation, and metastasis (68,69). Here, a balanced tensile strength facilitates attachment but also detachment as required for movement.

In this regard, it is remarkable that although the affinity of CD44 for HA as measured in conventional binding assays (i.e., without tensile stress; K_D between 10 and 100 μM, depending on the glycosylation state (70)) is lower than that of TSG-6 (~6 μM (36)) or HARE (<0.1 μM, E.N.H, unpublished data), its resistance to tensile forces is substantially higher (Table 1). This highlights that there is no strict correlation between affinity and resistance to mechanical stress, i.e., the hyaladherin family may have evolved such that these two interaction parameters can vary with a certain degree of independence.

What are the mechanical forces and loading rates exerted on individual HA•hyaladherin interactions in biological tissues? Currently, this question is difficult to answer because the supramolecular organization of HA-rich extracellular matrices (e.g., the intermolecular connectivity and the density of cross-links by multiple proteins) is largely unknown. Thus, even though we have a fairly good idea of the stresses and strains that various tissues experience, it is not known how these are distributed to the individual molecular bonds with the extracellular matrix. In this context, the here-presented quantitative data on the mechanical strength of individual HA•hyaladherin provide basic, molecular-level information that in the future can be fed into multiscale models of extracellular matrix mechanics that link molecular and tissue mechanics. Moreover, this also highlights the need for molecular probes that are able to measure molecular forces in tissues.

CONCLUSIONS

We have quantified the response of HA•hyaladherin bonds to tensile forces at the single bond level by applying a recently developed approach that is based on well-defined protein immobilization (validated by QCM-D) and AFM SMFS. We have measured distinct dynamic force spectra in which the mean unbinding forces vary approximately linearly with the logarithm of the instantaneous loading rate (consistent with the Bell-Evans model for bond rupture) for all HA•hyaladherin bonds tested. We have demonstrated that, within the range of loading rates probed (~10^2–10^4 pN/s), the bond of HA with AG1•LP (a complex of two type C hyaladherins) is mechanically more resilient than with VG1 (a single type C hyaladherin) and that the mean unbinding force decreases further for CD44 (type B hyaladherin) and HARE and TSG-6 (type A hyaladherins). These molecular-level data contribute to our mechanistic understanding of the mechanical properties of HA•hyaladherin complexes and HA-rich extracellular matrices and how these arise from their molecular composition and interactions.

SUPPORTING MATERIAL

Six figures and one table are available at http://www.biophysj.org/biophysj/supplemental/S0006-3495(18)30592-7.

AUTHOR CONTRIBUTIONS

R.P.R. conceived the research. F.B. and R.P.R. designed the experiments. F.B. performed the experiments. F.B. and R.P.R. analyzed the data and wrote the article. M.I.T., D.W.K., and E.N.H. provided essential reagents. All authors read and commented on the article.

ACKNOWLEDGMENTS

We thank L. Yate (CIC biomaGUNE) for gold deposition.

This work was supported by the European Research Council (starting grant "JELLY," FP7-ERC-2012-StG-306435, to R.P.R.), the United Kingdom Biotechnology and Biological Sciences Research Council (BB/R000174/1 to R.P.R.), the Spanish Ministry of Economy and Competitiveness (Retos grant MAT2014-54867-R to R.P.R.), and the National Institutes of Health (R01HL130864 to E.N.H).

REFERENCES

1. Day, A. J., and C. A. de la Motte. 2005. Hyaluronan cross-linking: a protective mechanism in inflammation? *Trends Immunol.* 26:637–643.
2. Evanko, S. P., M. I. Tammi, ..., T. N. Wight. 2007. Hyaluronan-dependent pericellular matrix. *Adv. Drug Deliv. Rev.* 59:1351–1365.
3. Jiang, D., J. Liang, and P. W. Noble. 2011. Hyaluronan as an immune regulator in human diseases. *Physiol. Rev.* 91:221–264.
4. Toole, B. P., T. N. Wight, and M. I. Tammi. 2002. Hyaluronan-cell interactions in cancer and vascular disease. *J. Biol. Chem.* 277:4593–4596.
5. Richter, R. P., N. S. Baranova, ..., J. C. Kwok. 2017. Glycosaminoglycans in extracellular matrix organisation: are concepts from soft matter physics key to understanding the formation of perineuronal nets? *Curr. Opin. Struct. Biol.* 50:65–74.
6. Han, L., A. J. Grodzinsky, and C. Ortiz. 2011. Nanomechanics of the cartilage extracellular matrix. *Annu. Rev. Mater. Res.* 41:133–168.
7. Morawski, M., G. Brückner, ..., R. T. Matthews. 2012. Aggrecan: beyond cartilage and into the brain. *Int. J. Biochem. Cell Biol.* 44:690–693.
8. Seror, J., Y. Merkher, ..., J. Klein. 2011. Articular cartilage proteoglycans as boundary lubricants: structure and frictional interaction of surface-attached hyaluronan and hyaluronan–aggrecan complexes. *Biomacromolecules.* 12:3432–3443.
9. Lee, R. T., C. Yamamoto, ..., T. N. Wight. 2001. Mechanical strain induces specific changes in the synthesis and organization of proteoglycans by vascular smooth muscle cells. *J. Biol. Chem.* 276:13847–13851.
10. Baranova, N. S., A. Inforzato, ..., R. P. Richter. 2014. Incorporation of pentraxin 3 into hyaluronan matrices is tightly regulated and promotes matrix cross-linking. *J. Biol. Chem.* 289:30481–30498.
11. McDonald, B., and P. Kubes. 2015. Interactions between CD44 and hyaluronan in leukocyte trafficking. *Front. Immunol.* 6:68.
12. Mohamadzadeh, M., H. DeGrendele, ..., M. Siegelman. 1998. Proinflammatory stimuli regulate endothelial hyaluronan expression and CD44/HA-dependent primary adhesion. *J. Clin. Invest.* 101:97–108.
13. Avigdor, A., P. Goichberg, ..., T. Lapidot. 2004. CD44 and hyaluronic acid cooperate with SDF-1 in the trafficking of human CD34+ stem/progenitor cells to bone marrow. *Blood.* 103:2981–2989.
14. Richter, U., D. Wicklein, ..., U. Schumacher. 2012. The interaction between CD44 on tumour cells and hyaluronan under physiologic flow

conditions: implications for metastasis formation. *Histochem. Cell Biol.* 137:687–695.

15. Harris, E. N., J. A. Weigel, and P. H. Weigel. 2004. Endocytic function, glycosaminoglycan specificity, and antibody sensitivity of the recombinant human 190-kDa hyaluronan receptor for endocytosis (HARE). *J. Biol. Chem.* 279:36201–36209.
16. Bano, F., S. Banerji, …, R. P. Richter. 2016. A single molecule assay to probe monovalent and multivalent bonds between hyaluronan and its key leukocyte receptor CD44 under force. *Sci. Rep.* 6:34176.
17. Raman, P. S., C. S. Alves, …, K. Konstantopoulos. 2012. Distinct kinetic and molecular requirements govern CD44 binding to hyaluronan versus fibrin(ogen). *Biophys. J.* 103:415–423.
18. Suzuki, T., M. Suzuki, …, I. Shimada. 2015. Mechanical force effect on the two-state equilibrium of the hyaluronan-binding domain of CD44 in cell rolling. *Proc. Natl. Acad. Sci. USA.* 112:6991–6996.
19. Blundell, C. D., N. T. Seyfried, and A. J. Day. 2004. Structural and functional diversity of hyaluronan-binding proteins. *In* Chemistry and Biology of Hyaluronan. H. G. Garg and C. A. Hales, eds. Elsevier Science Ltd., pp. 189–204.
20. Day, A. J., and G. D. Prestwich. 2002. Hyaluronan-binding proteins: tying up the giant. *J. Biol. Chem.* 277:4585–4588.
21. Kohda, D., C. J. Morton, …, A. J. Day. 1996. Solution structure of the link module: a hyaluronan-binding domain involved in extracellular matrix stability and cell migration. *Cell.* 86:767–775.
22. Higman, V. A., D. C. Briggs, …, A. J. Day. 2014. A refined model for the TSG-6 link module in complex with hyaluronan: use of defined oligosaccharides to probe structure and function. *J. Biol. Chem.* 289:5619–5634.
23. Day, A. J. 1999. The structure and regulation of hyaluronan-binding proteins. *Biochem. Soc. Trans.* 27:115–121.
24. Banerji, S., A. J. Day, …, D. G. Jackson. 1998. Characterization of a functional hyaluronan-binding domain from the human CD44 molecule expressed in Escherichia coli. *Protein Expr. Purif.* 14:371–381.
25. Watanabe, H., L. Gao, …, Y. Yamada. 1995. Mouse aggrecan, a large cartilage proteoglycan: protein sequence, gene structure and promoter sequence. *Biochem. J.* 308:433–440.
26. Wight, T. N. 2002. Versican: a versatile extracellular matrix proteoglycan in cell biology. *Curr. Opin. Cell Biol.* 14:617–623.
27. Seyfried, N. T., G. F. McVey, …, A. J. Day. 2005. Expression and purification of functionally active hyaluronan-binding domains from human cartilage link protein, aggrecan and versican: formation of ternary complexes with defined hyaluronan oligosaccharides. *J. Biol. Chem.* 280:5435–5448.
28. Banerji, S., A. J. Wright, …, D. G. Jackson. 2007. Structures of the Cd44-hyaluronan complex provide insight into a fundamental carbohydrate-protein interaction. *Nat. Struct. Mol. Biol.* 14:234–239.
29. Evans, E. 2001. Probing the relation between force–lifetime–and chemistry in single molecular bonds. *Annu. Rev. Biophys. Biomol. Struct.* 30:105–128.
30. Ott, W., M. A. Jobst, …, M. A. Nash. 2017. Single-molecule force spectroscopy on polyproteins and receptor-ligand complexes: the current toolbox. *J. Struct. Biol.* 197:3–12.
31. Harder, A., A. K. Möller, …, D. Anselmetti. 2015. Catch bond interaction between cell-surface sulfatase Sulf1 and glycosaminoglycans. *Biophys. J.* 108:1709–1717.
32. Sevim, S., S. Ozer, …, T. Lühmann. 2017. Nanomechanics on FGF-2 and heparin reveal slip bond characteristics with pH dependency. *ACS Biomater. Sci. Eng.* 3:1000–1007.
33. Tammi, R., U. M. Agren, …, M. Tammi. 1994. Hyaluronan metabolism in skin. *Prog. Histochem. Cytochem.* 29:1–81.
34. Jadin, L., L. Huang, …, H. M. Shepard. 2014. Characterization of a novel recombinant hyaluronan binding protein for tissue hyaluronan detection. *J. Histochem. Cytochem.* 62:672–683.
35. Harris, E. N., and P. H. Weigel. 2008. The ligand-binding profile of HARE: hyaluronan and chondroitin sulfates A, C, and D bind to overlapping sites distinct from the sites for heparin, acetylated low-density lipoprotein, dermatan sulfate, and CS-E. *Glycobiology.* 18:638–648.
36. Baranova, N. S., E. Nilebäck, …, R. P. Richter. 2011. The inflammation-associated protein TSG-6 cross-links hyaluronan via hyaluronan-induced TSG-6 oligomers. *J. Biol. Chem.* 286:25675–25686.
37. Dubacheva, G. V., C. Araya-Callis, …, R. P. Richter. 2017. Controlling multivalent binding through surface chemistry: model study on streptavidin. *J. Am. Chem. Soc.* 139:4157–4167.
38. Goldstein, B., D. Coombs, …, C. Wofsy. 1999. The influence of transport on the kinetics of binding to surface receptors: application to cells and BIAcore. *J. Mol. Recognit.* 12:293–299.
39. Takahashi, R., S. Al-Assaf, …, K. Nishinari. 2003. Asymmetrical-flow field-flow fractionation with on-line multiangle light scattering detection. 1. Application to wormlike chain analysis of weakly stiff polymer chains. *Biomacromolecules.* 4:404–409.
40. Reviakine, I., D. Johannsmann, and R. P. Richter. 2011. Hearing what you cannot see and visualizing what you hear: interpreting quartz crystal microbalance data from solvated interfaces. *Anal. Chem.* 83:8838–8848.
41. Butt, H. J., and M. Jaschke. 1995. Calculation of thermal noise in atomic force microscopy. *Nanotechnology.* 6:1–7.
42. Bustamante, C., J. F. Marko, …, S. Smith. 1994. Entropic elasticity of lambda-phage DNA. *Science.* 265:1599–1600.
43. Dudko, O. K., G. Hummer, and A. Szabo. 2008. Theory, analysis, and interpretation of single-molecule force spectroscopy experiments. *Proc. Natl. Acad. Sci. USA.* 105:15755–15760.
44. Williams, P. M. 2003. Analytical descriptions of dynamic force spectroscopy: behaviour of multiple connections. *Anal. Chim. Acta.* 479:107–115.
45. Evans, E., and K. Ritchie. 1997. Dynamic strength of molecular adhesion bonds. *Biophys. J.* 72:1541–1555.
46. Migliorini, E., D. Thakar, …, R. P. Richter. 2014. Well-defined biomimetic surfaces to characterize glycosaminoglycan-mediated interactions on the molecular, supramolecular and cellular levels. *Biomaterials.* 35:8903–8915.
47. Deisenhofer, J. 1981. Crystallographic refinement and atomic models of a human Fc fragment and its complex with fragment B of protein A from Staphylococcus aureus at 2.9- and 2.8-A resolution. *Biochemistry.* 20:2361–2370.
48. Attili, S., O. V. Borisov, and R. P. Richter. 2012. Films of end-grafted hyaluronan are a prototype of a brush of a strongly charged, semiflexible polyelectrolyte with intrinsic excluded volume. *Biomacromolecules.* 13:1466–1477.
49. Hermens, W. T., M. Benes, …, H. Speijer. 2004. Effects of flow on solute exchange between fluids and supported biosurfaces. *Biotechnol. Appl. Biochem.* 39:277–284.
50. Janshoff, A., M. Neitzert, …, H. Fuchs. 2000. Force spectroscopy of molecular systems-single molecule spectroscopy of polymers and biomolecules. *Angew. Chem. Int.Engl.* 39:3212–3237.
51. Fujii, T., Y. L. Sun, …, Z. P. Luo. 2002. Mechanical properties of single hyaluronan molecules. *J. Biomech.* 35:527–531.
52. Verbelen, C., H. J. Gruber, and Y. F. Dufrêne. 2007. The NTA-His6 bond is strong enough for AFM single-molecular recognition studies. *J. Mol. Recognit.* 20:490–494.
53. Pfreundschuh, M., D. Harder, …, D. J. Müller. 2017. Detecting ligand-binding events and free energy landscape while imaging membrane receptors at subnanometer resolution. *Nano Lett.* 17:3261–3269.
54. Schoeler, C., T. Verdorfer, …, M. A. Nash. 2016. Biasing effects of receptor-ligand complexes on protein-unfolding statistics. *Phys. Rev. E.* 94:042412.
55. Sedlak, S. M., M. S. Bauer, …, H. E. Gaub. 2017. Monodisperse measurement of the biotin-streptavidin interaction strength in a well-defined pulling geometry. *PLoS One.* 12:e0188722.
56. Liu, X., J. Q. Sun, …, Z. P. Luo. 2006. Force-mediated dissociation of proteoglycan aggregate in articular cartilage. *Biorheology.* 43:183–190.

57. Christophis, C., I. Taubert, …, A. Rosenhahn. 2011. Shear stress regulates adhesion and rolling of CD44+ leukemic and hematopoietic progenitor cells on hyaluronan. *Biophys. J.* 101:585–593.

58. Rees, S. G., C. L. Curtis, …, B. Caterson. 2005. Catabolism of aggrecan proteoglycan aggregate components in short-term explant cultures of tendon. *Matrix Biol.* 24:219–231.

59. Rodriguez, E., and P. Roughley. 2006. Link protein can retard the degradation of hyaluronan in proteoglycan aggregates. *Osteoarthritis Cartilage.* 14:823–829.

60. Bayliss, M. T., S. Howat, …, J. Dudhia. 2000. The organization of aggrecan in human articular cartilage. Evidence for age-related changes in the rate of aggregation of newly synthesized molecules. *J. Biol. Chem.* 275:6321–6327.

61. Knudson, W., S. Ishizuka, …, C. B. Knudson. 2018. The pericellular hyaluronan of articular chondrocytes. *Matrix Biol* Published online February 6, 2018. https://doi.org/10.1016/j.matbio.2018.02.005.

62. Verzijl, N., J. DeGroot, …, J. M. TeKoppele. 2001. Age-related accumulation of the advanced glycation endproduct pentosidine in human articular cartilage aggrecan: the use of pentosidine levels as a quantitative measure of protein turnover. *Matrix Biol.* 20:409–417.

63. Falkowski, M., K. Schledzewski, …, S. Goerdt. 2003. Expression of stabilin-2, a novel fasciclin-like hyaluronan receptor protein, in murine sinusoidal endothelia, avascular tissues, and at solid/liquid interfaces. *Histochem. Cell Biol.* 120:361–369.

64. Harris, E. N., S. V. Kyosseva, …, P. H. Weigel. 2007. Expression, processing, and glycosaminoglycan binding activity of the recombinant human 315-kDa hyaluronic acid receptor for endocytosis (HARE). *J. Biol. Chem.* 282:2785–2797.

65. Harris, E. N., J. A. Weigel, and P. H. Weigel. 2008. The human hyaluronan receptor for endocytosis (HARE/Stabilin-2) is a systemic clearance receptor for heparin. *J. Biol. Chem.* 283:17341–17350.

66. Blundell, C. D., A. Almond, …, A. J. Day. 2005. Towards a structure for a TSG-6.hyaluronan complex by modeling and NMR spectroscopy: insights into other members of the link module superfamily. *J. Biol. Chem.* 280:18189–18201.

67. Ponta, H., L. Sherman, and P. A. Herrlich. 2003. CD44: from adhesion molecules to signalling regulators. *Nat. Rev. Mol. Cell Biol.* 4:33–45.

68. Bourguignon, L. Y., M. Shiina, and J. J. Li. 2014. Hyaluronan-CD44 interaction promotes oncogenic signaling, microRNA functions, chemoresistance, and radiation resistance in cancer stem cells leading to tumor progression. *Adv. Cancer Res.* 123:255–275.

69. Puré, E., and R. K. Assoian. 2009. Rheostatic signaling by CD44 and hyaluronan. *Cell. Signal.* 21:651–655.

70. Banerji, S., B. R. Hide, …, D. G. Jackson. 2010. Distinctive properties of the hyaluronan-binding domain in the lymphatic endothelial receptor Lyve-1 and their implications for receptor function. *J. Biol. Chem.* 285:10724–10735.

71. Roughley, P. J. 2006. The structure and function of cartilage proteoglycans. *Eur. Cell. Mater.* 12:92–101.

72. Harris, E. N. 2012. Heparin clearance by liver scavenger receptors. *Biochem. Anal. Biochem.* 1:e114.

Biophysical Journal, Volume *114*

Supplemental Information

Single-Molecule Unbinding Forces between the Polysaccharide Hyaluronan and Its Binding Proteins

Fouzia Bano, Markku I. Tammi, David W. Kang, Edward N. Harris, and Ralf P. Richter

SUPPORTING FIGURES

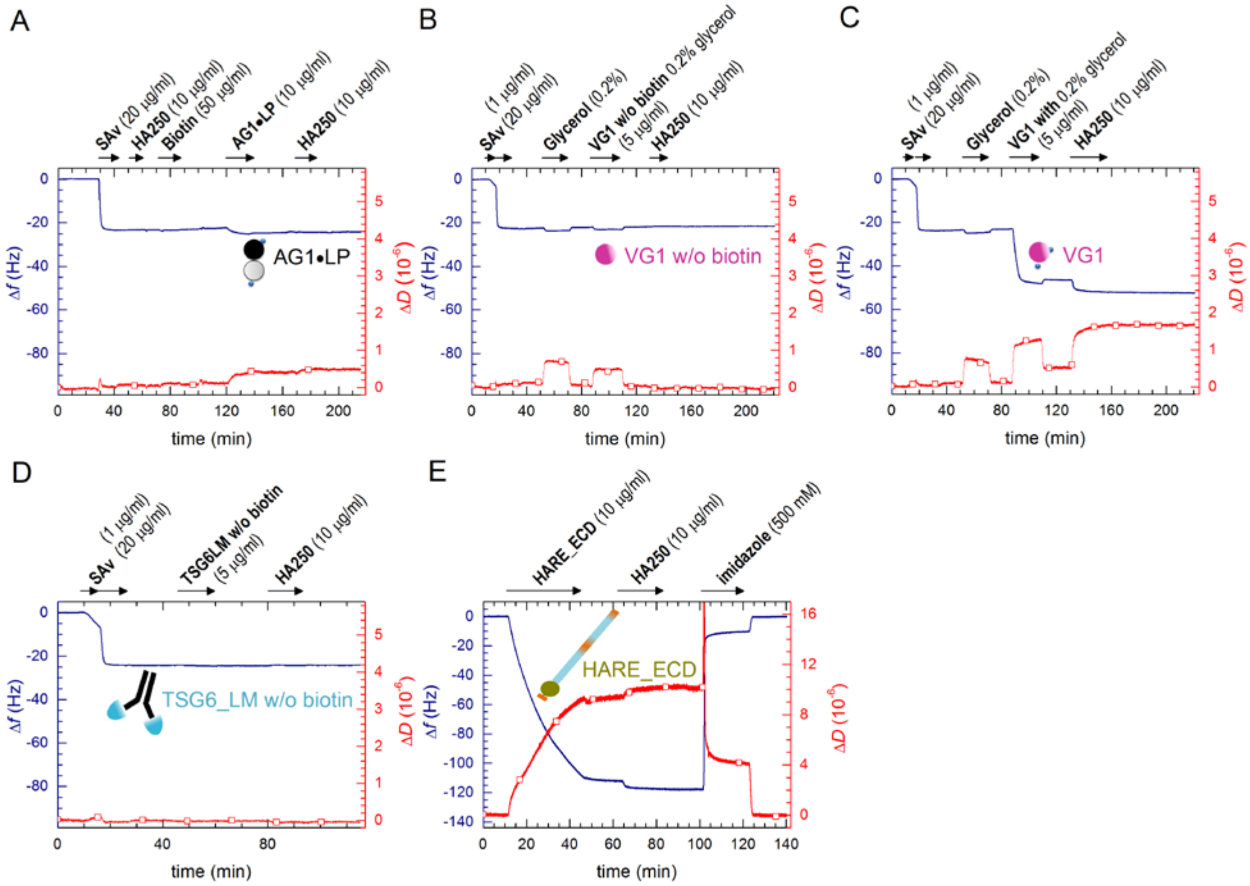

Figure S1: Complementary QCM-D assays demonstrating the specificity of hyaladherin immobilization and HA binding. Data are presented analogous to Fig. 2. (A-D) Streptavidin monolayers were either blocked with free biotin prior to hyaladherin incubation (for AG1•LP; A) or exposed to hyaladherins lacking biotin tags (for VG1 and TSG6_LM; B and D, respectively). In combination with Fig. 2, the QCM-D responses shown here demonstrate non-specific binding of only a minor fraction of AG1•LP (A) and no detectable non-specific binding for VG1 (B) and TSG6_LM (D), and that HA binds exclusively to the specifically immobilized proteins. B and C also contain an incubation step of the streptavidin monolayer with 0.2% glycerol in working buffer (Δf = -1.5 ± 0.4 Hz and ΔD = 0.6 ± 0.1 × 10^{-6}), a concentration that is also contained in the working solutions of incubated VG1. This control demonstrates that the QCM-D responses for VG1 without biotin (B) are due to the presence of glycerol and do not reflect a transient binding, and that the responses for VG1 with biotin (C) are also affected by glycerol. This solution effect has been corrected for in Fig. 2B. (E) HARE_ECD can be fully eluted in imidazole, demonstrating that binding to the His-tag-capturing layer is specific through the His tag. Changes in Δf and ΔD upon exchange from imidazole containing solution to pure working buffer (at 123 min) do not reflect any changes on the surface but result from a change in the viscosity and/or density of the surrounding solution owing to the presence of imidazole. Similarly, the small decrease in dissipation (ΔD = 0.4 ± 0.1 × 10^{-6}) upon exchange from HARE_ECD containing solution to pure working buffer (at 46 min) reflects the effect of residual buffer components in the protein stock solution, and this has been corrected for in Fig. 2D.

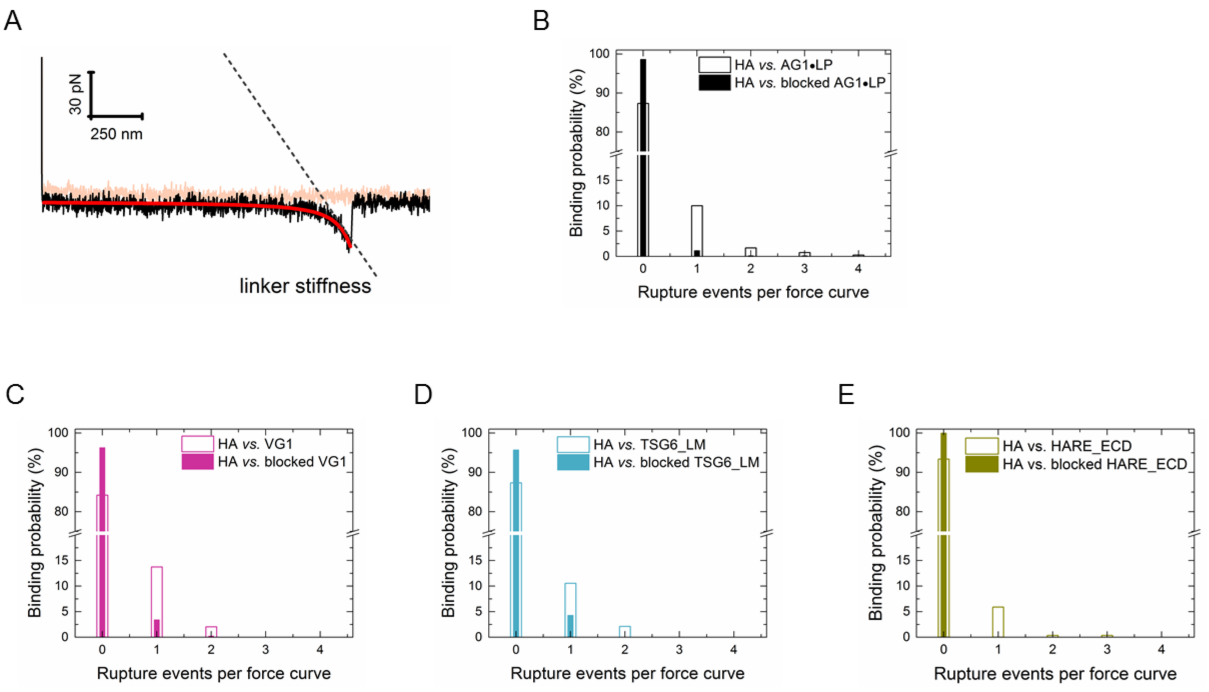

Figure S2: (A) A representative force curve (approach in pink and retract in black) acquired for a specific unbinding event between HA and VG1 (retract velocity 1500 nm/s). The solid red line is a best-fit WLC model curve for stretching a single HA chain. The slope of the dashed straight line represents the linker stiffness (*i.e.*, the slope of WLC fit) at bond rupture, which together with the retract velocity gives the instantaneous loading rate. (B-E) Probability of specific rupture events per force curve for the interactions as indicated in the plots (retract velocities: 2000 nm/s - AG1•LP, 3000 nm/s - VG1, 1000 nm/s - TSG6_LM and HARE_ECD). Competition with a low-molecular-weight HA polymer in solution (58 kDa; 10 µg/ml; filled bars) drastically reduced the occurrence of specific rupture events compared to the equivalent system without the competitor (open bars). The analysis is based on 1200/800 (AG1•LP; B), 342/400 (VG1; C), 237/200 (TSG6_LM; D) and 559/232 (HARE_ECD; E) force curves per sample for the measurements without/with competitor, respectively.

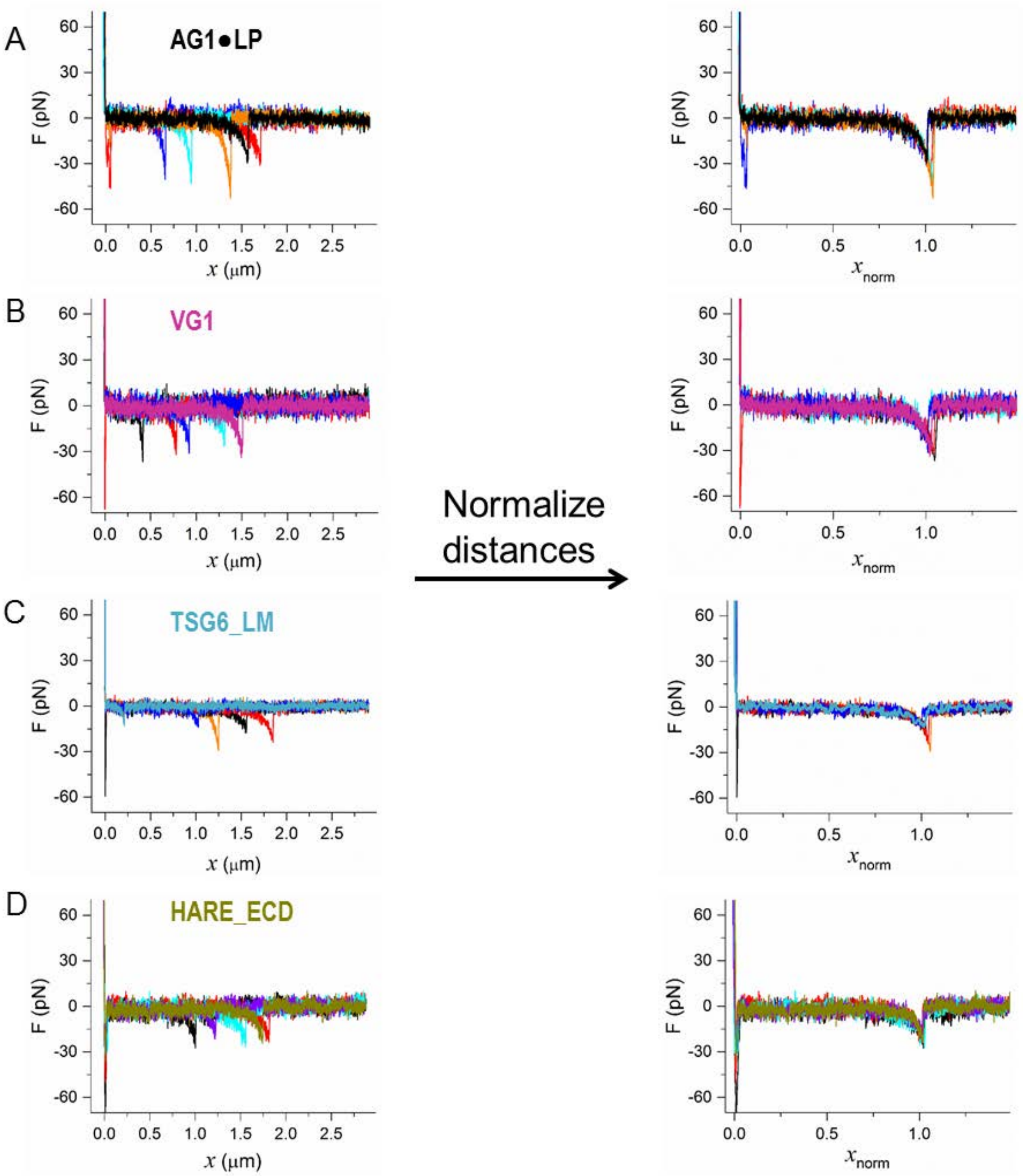

Figure S3: Comparison of force curves by normalization to account for variations in the locus of hyaladherin binding along the HA chain. Shown on the left are sets of 5 force curves per hyaladherin (as indicated; retract velocity 1000 nm/s) featuring a single specific rupture event along with non-specific interactions at very small separations. Curves of force *F* *vs.* distance *x* were selected at random but with a bias to cover a large spectrum of binding loci on the HA chain; that is, the distances at rupture (ranging from 245 to 1852 nm) span a large part of the HA contour length (2.1 μm). Shown on the right are the same force curves with the distances re-scaled such that $x_{norm} = x/x^*$ with $F(x^*) = F^*$ where the force F^* was set individually for each hyaladherin to be slightly inferior to the rupture forces (AG1•LP – 24 pN, VG1 – 19 pN, TSG6_LM – 10 pN, HARE_ECD – 13 pN). According to the WLC model, parts of the curves prior to the rupture event should overlap if the stretching and unbinding of a single HA chain is consistently being probed (1), and this is indeed the case.

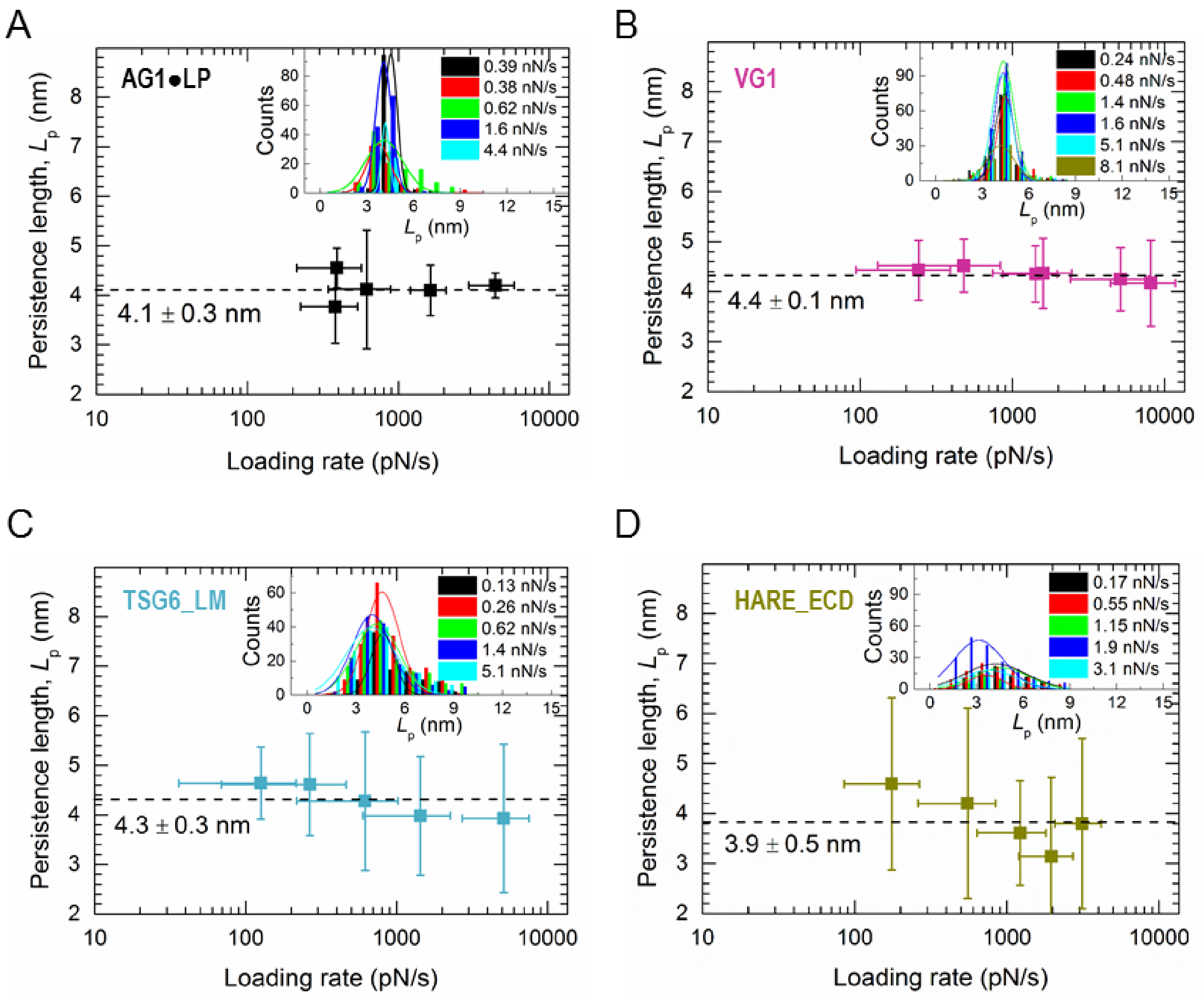

Figure S4: Persistence length (L_p) versus instantaneous loading rates for the data shown in Fig. 4. Dashed horizontal lines represent the mean and numbers indicate the mean ± s.d. The inset shows histograms of L_p for the studied instantaneous loading rates (as listed with color codes) with Gaussian fits from which the mean and standard deviations were calculated. From the four mean values displayed in A to D, a mean persistence length of 4.2 ± 0.2 nm (mean ± s.d.) was extracted.

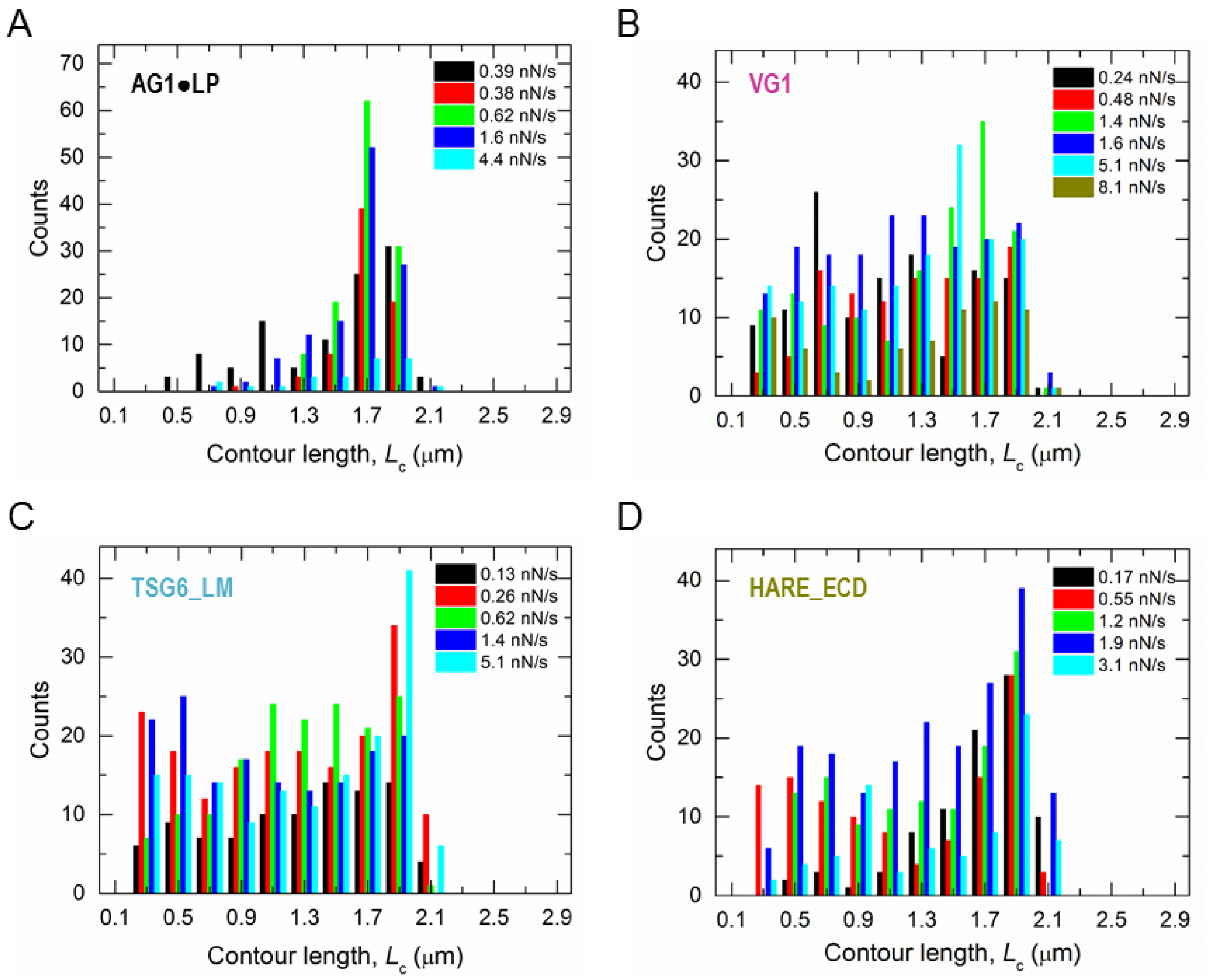

Figure S5: Histograms of contour lengths for the data shown in Fig. 4, displayed by loading rates (as listed with color codes).

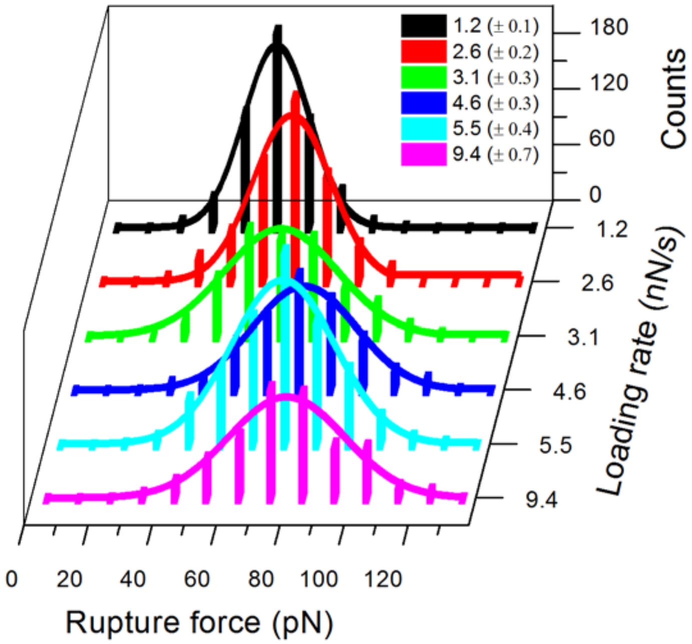

Figure S6: Rupture force histograms for various instantaneous loading rates (listed with color codes as mean ± s.d.) for the interaction of streptavidin and biotin. Solid lines represent Gaussian fits. Data adapted from (2), where streptavidin was immobilized as described in the present work and biotin was tethered *via* a thiol-poly(ethylene glycol) to a gold-coated AFM tip.

SUPPORTING TABLES

Table S1: Overview of force curve sample sizes.

HA binding to	AFM probe	Mean loading rate (nN/s)	Number of force curves			
			Total	Specific rupture events		
				0	1	≥2
AG1•LP	NPG	0.38	3159	2945	106	54
	OBL	0.39	1001	931	58	6
	NPG	0.62	1027	910	90	27
	NPG	1.6	1001	919	50	32
	NPG	4.4	603	577	23	3
VG1	OBL	0.24	708	582	121	5
		0.48	784	644	113	27
		1.4	1367	1182	147	33
		1.6	564	386	145	33
		5.1	903	729	156	18
		8.1	1429	1357	69	3
TSG6_LM	OBL	0.13	778	698	64	16
		0.26	1293	185	127	29
		0.62	757	630	93	34
		1.4	703	572	105	26
		5.1	775	663	97	15
HARE_ECD	OBL	0.17	1442	1355	67	20
		0.55	1001	893	85	23
		1.2	1485	1364	92	29
		1.9	1395	1240	127	28
		3.1	1216	1141	61	14

SUPPORTING REFERENCES

1. Janshoff, A., M. Neitzert, Y. Oberdorfer, and H. Fuchs. 2000. Force Spectroscopy of Molecular Systems-Single Molecule Spectroscopy of Polymers and Biomolecules. Angew. Chem. Int. Ed. Engl. 39:3212-3237.
2. Bano, F., S. Banerji, M. Howarth, D. G. Jackson, and R. P. Richter. 2016. A single molecule assay to probe monovalent and multivalent bonds between hyaluronan and its key leukocyte receptor CD44 under force. Sci. Rep. 6:34176.
